# Negative Affect Circuit Subtypes and Neural, Behavioral, and Affective Responses to MDMA: A Randomized Clinical Trial

**DOI:** 10.1001/jamanetworkopen.2025.7803

**Published:** 2025-04-30

**Authors:** Xue Zhang, Laura M. Hack, Claire Bertrand, Rachel Hilton, Nancy J. Gray, Leyla Boyar, Jessica Laudie, Boris D. Heifets, Trisha Suppes, Peter J. van Roessel, Carolyn I. Rodriguez, Karl Deisseroth, Brian Knutson, Leanne M. Williams

**Affiliations:** 1 Department of Psychiatry and Behavioral Sciences, Stanford University School of Medicine, Stanford, California; 2 Sierra-Pacific Mental Illness Research, Education and Clinical Center, Veterans Affairs Palo Alto Health Care System, Palo Alto, California; 3 Department of Anesthesiology, Perioperative and Pain Medicine, Stanford University School of Medicine, Stanford, California; 4 Veterans Affairs Palo Alto Health Care System, Palo Alto, California; 5 Department of Bioengineering, Stanford University, Stanford, California; 6 Howard Hughes Medical Institute, Stanford University, Stanford, California; 7 Department of Psychology, Stanford University, Stanford, California

## Abstract

**Importance:**

Rapidly acting therapeutics like 3,4-methylenedioxymethamphetamine (MDMA) are promising treatments for disorders such as posttraumatic stress disorder (PTSD). However, understanding who benefits most and the underlying neural mechanisms remains a critical gap. Stratifying individuals by neural circuit profiles could help differentiate neural, behavioral, and affective responses to MDMA, enabling personalized treatment strategies.

**Objective:**

To investigate whether baseline stratification of individuals based on negative affect circuit profiles, particularly in response to nonconscious threat stimuli, can differentiate acute responses to MDMA.

**Design, Setting, and Participants:**

This randomized clinical trial, implementing a double-blinded, within-participant, placebo- and baseline-controlled design, was conducted at Stanford University School of Medicine between November 2, 2021, and November 9, 2022, for wave 1 data collection. Participants had used MDMA on at least 2 prior occasions, but not in the past 6 months, and had subthreshold PTSD symptoms and early life trauma but no current psychiatric disorders. Data were analyzed from March 1, 2023, to January 1, 2024.

**Interventions:**

Participants completed 4 visits: 1 baseline session followed by 1 placebo session and 2 MDMA sessions in a randomized order, totaling 64 visits. Baseline functional magnetic resonance imaging (fMRI) assessed the negative affect circuit using a nonconscious threat processing task (NTN).

**Main Outcomes and Measures:**

Primary outcomes included activity and connectivity of amygdala and subgenual anterior cingulate cortex (sgACC) defining the negative affect circuit. Secondary outcomes were behavioral measures of implicit threat bias, likability of threat expressions, and affective assessments.

**Results:**

Sixteen participants (10 [63%] female; mean [SD] age, 40.8 [7.6] years) were stratified into subgroups with high and low levels of NTN activity in the amygdala (NTN_A+_ [n = 8] and NTN_A−_ [n = 8], respectively), based on a median split of baseline nonconscious threat-evoked fMRI responses. Following administration of the 120 mg of MDMA vs placebo, the NTN_A+_ subgroup showed significant reductions in amygdala (contrast estimate [CE], −1.43; 95% CI, −2.60 to −0.27; Cohen *d*, −1.22; *P* = .02) and sgACC activity (CE, −1.48; 95% CI, −2.42 to −0.54; Cohen *d*, −1.56; *P* = .004), increased sgACC-amygdala connectivity (CE, 0.65; 95% CI, 0.02-1.28; Cohen *d*, 1.02; *P* = .04), and increased likability of threat expressions (CE, 14.38; 95% CI, 1.46-27.29; Cohen *d*, 0.86; *P* = .03) compared with the NTN_A−_ subgroup.

**Conclusions and Relevance:**

In this randomized clinical trial of MDMA’s acute profiles, 120 mg of MDMA acutely normalized negative affect circuit reactivity in participants stratified by heightened amygdala reactivity at baseline, demonstrating the potential of neuroimaging to identify prospective biomarkers and guide personalized MDMA-based therapies.

**Trial Registration:**

ClinicalTrials.gov Identifier: NCT04060108

## Key Points

**Question:**

Can baseline stratification of the amygdala-defined negative affect circuit differentiate acute neural, behavioral, and affective responses to 3,4-methylenedioxymethamphetamine (MDMA)?

**Findings:**

In this randomized clinical trial of 16 participants, those stratified to the high negative affect circuit activity subgroup demonstrated a regularized circuit response evoked by nonconscious threat following administration of 120 mg of MDMA vs placebo, along with improved appraisal of threat expressions.

**Meaning:**

These findings suggest that baseline stratification of negative affect circuit activity offers a potential biomarker for acute neural, behavioral, and affective effects of MDMA, underscoring the need for clinical studies to assess its relevance to therapeutic outcomes and inform personalized MDMA-based therapies.

## Teaser

This randomized clinical trial assesses the response of the negative affect circuit to implicit threat following acute administration of 3,4-methylenedioxymethamphetamine (MDMA) or placebo in adults with posttraumatic stress disorder.

## Introduction

Posttraumatic stress disorder (PTSD) affects millions worldwide and frequently co-occurs with major depression disorder (MDD), contributing significantly to disability and increased mortality.^1,2^ Despite significant clinical and neurobiological heterogeneity of PTSD and the availability of therapeutic options with different mechanisms of action,^3,4^ treatments are often prescribed through a “one-size-fits-all” trial-and-error approach. There is a critical need for mechanistic tests to precisely target the underlying causes and subtypes of PTSD to guide the development of more effective therapeutic strategies. Precision medicine in mental health seeks to fill this gap by tailoring treatments to each individual’s unique neurobiological profile, leveraging advances in human neuroimaging.^5,6,7,8,9^

Stratifying individuals into subgroups based on neural circuit profiles identified through neuroimaging offers a novel approach to advancing personalized mental health treatments.^10,11^ Functional magnetic resonance imaging (fMRI) studies have consistently shown amygdala hyperactivity in PTSD during the nonconscious processing of threat.^12,13^ Both positive and negative associations between threat-evoked amygdala hyperactivity and the ventral anterior cingulate cortex, including the subgenual region (sgACC), have been reported in PTSD,^13,14^ suggesting the existence of distinct subgroups within PTSD. The amygdala plays a central role in the negative affect circuit^15^ and is thought to be implicitly regulated by the sgACC.^16^ Hyperactivity in the amygdala following trauma, coupled with impaired sgACC regulation, may be most effectively revealed by threat stimuli presented outside conscious awareness. We highlight that amygdala hyperactivity during nonconscious threat processing has also been observed in MDD,^17^ which co-occurs in approximately half of individuals with PTSD.^18^ This hyperactivity has been linked to poor treatment outcomes in both PTSD and MDD, particularly for selective serotonin reuptake inhibitors and cognitive behavioral therapy.^19,20^ Consequently, targeting mechanisms within the negative affect circuit presents a promising avenue for developing precision treatments tailored to individuals with threat reactivity subtypes who do not respond to first-line therapies.

3,4-Methylenedioxymethamphetamine (MDMA) is a psychoactive entactogen known for its unique ability to reduce threat responses and promote positive emotional experiences.^21^ This characteristic presents a novel opportunity to examine its effects on modulating the negative affect circuit. MDMA-assisted therapy has shown promise in clinical settings, particularly for treating treatment-resistant PTSD.^22,23^ MDMA-assisted therapy leverages the acute pharmacological effects of MDMA to create a window of tolerance, allowing for emotional engagement with threat reactivity. This occurs within a structured protocol and a supportive environment, which together facilitate the reprocessing and reappraisal of trauma-related memories and events.^24^ In an fMRI study, MDMA-assisted therapy has been associated with increased resting functional connectivity between the amygdala and hippocampus at 2-month follow-up in individuals with PTSD, which may reflect therapeutic engagement of appraisal mechanisms.^25^ The specific neural mechanisms through which MDMA alone exerts its effects, particularly following acute administration, remain unclear. One prior fMRI study of nonclinical participants^26^ demonstrated that MDMA acutely attenuated amygdala activity in response to threat stimuli, which may reflect mechanisms of reduced threat reactivity.

We conducted a systematic fMRI investigation of the neural mechanisms that underlie the acute effects of MDMA administration, building on prior research with several innovative approaches. Using a within-participants, randomized, placebo- and baseline-controlled multimodal design, we examined the effects of placebo and 80- and 120-mg doses of MDMA hydrochloride in adult participants with varying levels of PTSD symptoms and histories of early life trauma (ie, nonclinical). Our study assessed the response of the negative affect circuit to implicit threat following acute MDMA or placebo administration. A key strength of our design was the inclusion of a true baseline visit, distinct from the placebo and MDMA conditions, conducted prior to any drug administration. This allowed us to accurately account for baseline neural activity before any intervention. As preregistered in our study protocol (Supplement 1), we examined whether MDMA-induced changes in neural, behavioral, and affective profiles were dependent on baseline stratification of the response of the negative affect circuit to implicit threat. We hypothesized that following acute MDMA administration, individuals with higher baseline activity would show significant normalization across neural, behavioral, and affective measures compared with those with lower activity.

## Methods

This study was approved by the Stanford University Institutional Review Board and was preregistered on August 6, 2019. All participants provided written informed consent. Details of the trial protocol, participants, visits, and assessments are presented in Supplement 1 (trial protocol) and eMethods in Supplement 2. This study followed the Consolidated Standards of Reporting Trials (CONSORT) reporting guideline.

### Participants

**Figure 1.**
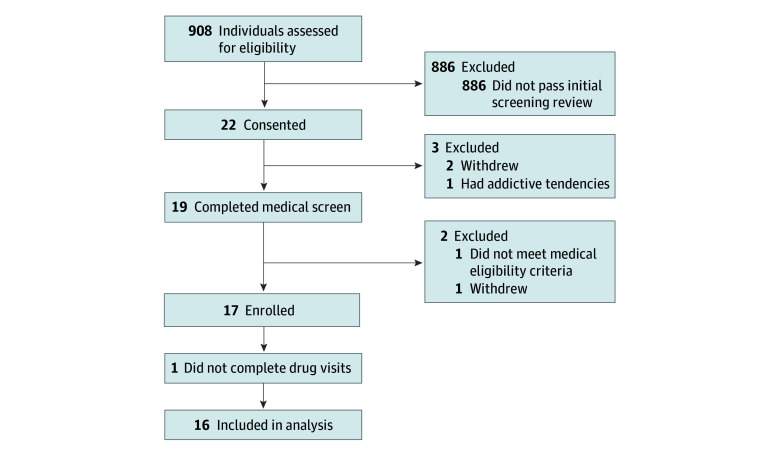
Participant Flow Diagram

Seventeen adults (aged 18-55 years) were recruited from the community (Figure 1). Primary inclusion criteria included at least 2 prior MDMA uses after 18 years of age, but not within 6 months of the first study dose; no current mood, anxiety, or substance use disorder; and no current or past eating, bipolar, or psychotic disorder. Participants refrained from use of psychoactive substances, supplements, and nonprescription medications during the study, with compliance confirmed by urine drug test results at screening and each drug visit (for a full list of inclusion and exclusion criteria, see the Box).

Box. **Inclusion and Exclusion Criteria**

**Inclusion criteria**

Aged 18-55 years
All genders and ethnic and racial categories
At least 2 prior uses of MDMA when 18 years or older and reported no serious adverse reactions from MDMA or ecstasy
Able to swallow capsules
Able to receive an MRI
Able to comply with study procedures
Able and willing to enroll and provide written informed consent

**Exclusion criteria**

Current mood or anxiety disorder
Current or past eating, bipolar, or psychotic disorder
Current substance or alcohol use disorder
Current suicide risk
Schizophrenia in a first-degree relative
Allergy or hypersensitivity to MDMA
Have used ecstasy or MDMA within 6 months of the first study dose or have previously participated in a MAPS-sponsored MDMA clinical trial
Concurrent use of any medication or substance that might increase the risk of participation and/or interaction with MDMA
Current use of any psychotropic medication^a^
Unable or unwilling to agree to refrain from using any psychoactive substances, supplements, and nonprescription medications starting 1 week prior to study start and for the duration of study
Unable or unwilling to refrain from using caffeine for 12 hours before and 10 hours after drug administration
Direct physical access to or routinely handling of addicting drugs in the regular course of work duties
Current use of any opioids, including codeine, hydrocodone, and morphine
Positive test result on urine drug screen for illicit drugs and/or drugs of abuse at screening and prior to study drug administration
BMI outside of healthy range (18-30)
Pregnant or nursing
Kidney or hepatic impairment
Uncontrolled hypertension^b^
Bradycardia or tachycardia^c,d^
Current chronic congestive heart failure, tachyarrhythmias, or myocardial ischemia
Marked baseline prolongation of QT or QTc interval
Current or history of significant (controlled or uncontrolled) hematological, endocrine, cerebrovascular, cardiovascular, coronary, pulmonary, kidney, gastrointestinal, immunocompromising, or neurological disease
History of additional risk factors for Torsade de pointes
History of hyponatremia or hyperthermia
Inability to speak, read, or understand English at a fifth grade level or severe hearing impairment
Plan to move out of the area during the study period
An exclusionary metal device

Abbreviations: BMI, body mass index (calculated as weight in kilograms divided by the height in meters squared); MAPS, Multidisciplinary Association for Psychedelic Studies; MDMA, 3,4-methylenedioxymethamphetamine; MRI, magnetic resonance imaging.

^a^
If the participant reports recently discontinuing a psychotropic medication, a washout period of 5 half-lives will be required prior to drug visits followed by a 1-week stabilization period.

^b^
Hypertension defined by systolic blood pressure of at least 140 mm Hg and/or diastolic blood pressure of at least 90 mm Hg.

^c^
Bradycardia defined by heart rate less than 50 bpm.

^d^
Tachycardia defined by heart rate greater than 150 bpm.

### Study Design

In this double-blinded, within-participants, placebo- and baseline-controlled randomized clinical trial, each participant completed 4 visits: a baseline visit and 3 subsequent drug visits in a randomized order (matched placebo, 80-mg dose of MDMA hydrochloride, or 120-mg dose of MDMA hydrochloride 10-14 days apart) (see eFigure 1 in Supplement 2 for the study design). During the drug visits, participants, the research coordinators (C.B., N.J.G., L.B., and J.L.), and licensed study clinicians (L.M.H., R.H., B.D.H., and P.J.v.R.) were masked to the intervention, and masking was assessed by asking them to guess the dose at the end of each visit. The inclusion of 80-mg as well as 120-mg doses offered the opportunity to evaluate whether the acute effects of MDMA on neural, behavioral, and subjective affective responses are distinguished by these doses, while also enhancing masking by making it more difficult for participants to distinguish between doses.

### Primary Outcomes: Negative Affect Circuit Activity and Connectivity

The nonconscious Facial Expressions of Emotion Task was used to assess nonconscious threat-evoked activity and connectivity of amygdala and sgACC at the baseline visit and all drug visits at approximately 90 minutes after administration. Neural activity and connectivity evoked by threat vs neutral faces were expressed in *z* scores relative to a healthy reference group (n = 50). Data acquisition, preprocessing, and region of interest definitions are detailed in the eMethods in Supplement 2.

### Secondary Outcomes: Behavioral and Affective Measures

Behavior implicit emotion bias was assessed using the WebNeuro implicit recognition of facial emotions test after the scan at the baseline visit and all drug visits.^27,28^ Implicit threat bias was operationalized as reaction times for angry minus neutral faces, standardized (as *z* scores) against age-matched norms.^29^ At all drug visits, participants also completed a facial likability assessment, rating the likability of faces seen during the fMRI task (scale of 0-100) after the scan. Affective responses were measured after the scan using a visual analog scale (VAS) and the Five-Dimensional Altered States of Consciousness (5D-ASC) scale. Key VAS items (“I want to be with other people” and “I feel secure”) and 5D-ASC items (Anxiety and Impaired Control and Cognition) assessed MDMA-induced positive and negative affective states, respectively. Narrative descriptions of participant experiences were also recorded.

### Baseline Stratification and Validation by Negative Affect Circuit Activity Levels

All analyses were conducted in R, version 4.1.3, using R Studio, version 1.4.1106 (R Program for Statistical Computing). Participants were stratified into subgroups with high and low levels of negative affect circuit activity (NTN_A+_ and NTN_A−_, respectively) based on mean amygdala activity in response to nonconscious threat at baseline using a median split (eFigure 1 in Supplement 2). NTN refers to the negative affect circuit evoked by nonconscious threat stimuli, while the subscript A+ and A− indicate hyperactivity (high) and hypoactivity (low) following the nomenclature from previous work by Tozzi et al.^17^ Stratification was validated behaviorally by the implicit emotion bias, using a 2-sample *t* test. Baseline demographics and clinical symptoms were analyzed for potential contributions to stratification using 2-sample *t* tests or χ^2^ tests. Demographics included self-reported race, collected to describe our study population and included in the analysis to assess its potential contribution to stratification.

### Statistical Analysis

Data were analyzed from March 1, 2023, to January 1, 2024. To evaluate the differentiating value of baseline stratification on primary and secondary outcomes, linear mixed-effects models were implemented with baseline subgroup (NTN_A+_ or NTN_A−_), dose (placebo, 80 mg of MDMA, or 120 mg of MDMA), and subgroup-by-dose interaction as fixed effects, using the nlme package of R, version 1.1.162.^30^ Covariates, including age, biological sex, and the percentage of motion spikes, were included if they were found to associate with outcomes. We focused on the between-subgroup difference between the placebo and the 120-mg MDMA dose, as well as between the placebo and the 80-mg MDMA dose. Effect sizes were calculated as Cohen’s *d* using the effectsize package (R, version 0.8.3).^31^ Statistical significance was defined as a 2-sided *P* < .05.

## Results

Sixteen participants (mean [SD] age, 40.8 [7.59] years; 10 female [63%] and 6 male [38%]; 1 [6%] American Indian or Alaska Native, 1 [6%] Asian, 1 [6%] Black or African American, 12 [75%] White, and 1 [6%] multiracial) with a range of subthreshold PTSD symptoms and early life traumatic events were recruited into this MDMA trial and completed all 4 visits (Table). They were stratified into NTN_A+_ and NTN_A−_ subgroups using a median split of the amygdala in response to nonconscious threat at baseline, which resulted in an equal number of participants in each subgroup (n = 8).

**Table.** Demographic Characteristics and Baseline Clinical Symptoms

Measure	No. (%) of participants		
	All (n = 16)	NTN_A+_ (n = 8)	NTN_A−_ (n = 8)
Demographic			
Age, mean (SD), y	40.8 (7.59)	41.8 (9.05)	39.8 (6.27)
Biological sex			
Male	6 (38)	3 (38)	3 (38)
Female	10 (63)	5 (63)	5 (63)
Educational level			
4-y College	9 (56)	4 (50)	5 (63)
More than 4-y college	7 (44)	4 (50)	3 (38)
Handedness			
Right	15 (94)	8 (100)	7 (88)
Left	0	0	0
Ambidextrous	1 (6)	0	1 (13)
Race			
American Indian or Alaska Native	1 (6)	1 (13)	0
Asian	1 (6)	0	1 (13)
Black or African American	1 (6)	0	1 (12)
White	12 (75)	6 (75)	6 (75)
Multiracial	1 (6)	1 (13)	0
PCL-C, mean (SD)^a^	20.4 (2.33)	20.9 (1.81)	19.9 (2.80)
PHQ-9, mean (SD)^b^	1.38 (1.02)	1.63 (1.06)	1.13 (0.991)
GAD-7, mean (SD)^c^	1.38 (1.96)	1.25 (2.12)	1.50 (1.93)
Adverse childhood events, mean (SD)^d^	2.87 (2.45)	3.71 (1.89)	2.13 (2.75)

Abbreviations: GAD-7, 7-item Generalized Anxiety Disorder; NTN_A+_, high negative affect circuit activity evoked by nonconscious threat; NTN_A−_, low negative affect circuit activity evoked by nonconscious threat; PCL-C, Post-Traumatic Stress Disorder Checklist–Civilian Version; PHQ-9, 9-Item Patient Health Questionnaire.

^a^ Scores range from 17 to 85, with higher scores indicating a greater severity of posttraumatic stress disorder symptoms.

^b^ Scores range from 0 to 27, with higher scores indicating greater depression severity.

^c^ Scores range from 0 to 21, with higher scores indicating greater severity of anxiety.

^d^ Measured by the Early Life Stress Questionnaire. Scores range from 0 to 19, with higher scores indicating greater exposure to childhood trauma.

### Baseline Stratification and Validation Into Subgroups

**Figure 2.**
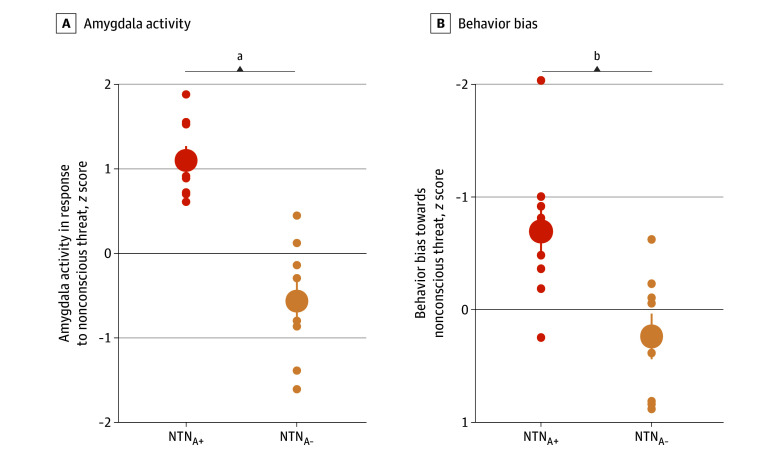
Baseline Stratification and Validation of the High (NTN_A+_) or Low (NTN_A-_) Negative Affect Circuit Activity Subgroups Defined by Amygdala Activity Evoked by Nonconscious Threat A, At the baseline visit, each participant was assessed by the negative affect circuit during facial expressions of emotion functional magnetic resonance imaging (fMRI) task, in which participants nonconsciously viewed facial expressions of emotion. Activity of the amygdala in response to threat faces relative to neutral faces was quantified and expressed in SD units relative to a separate healthy reference dataset (*z* scores). A median split was used to stratify all participants into NTN_A+_ (n = 8) and NTN_A−_ (n = 8) negative affect circuit activity subgroups. Validating the stratification, the NTN_A+_ subgroup had mean activity significantly above the mean of the healthy reference (mean *z* score, 1.09), while the NTN_A−_ subgroup had mean activity significantly below the mean of the healthy reference (mean *z* score, −0.57). B, Compared with the NTN_A−_ subgroup, the NTN_A+_ subgroup had a nonconscious implicit bias toward threat at baseline, indicated by slowed reaction times on an implicit recognition test when primed by angry relative to neutral faces using the same facial expression stimuli as presented during the fMRI task. This implicit priming reaction time measure was also expressed as *z* score SD units relative to healthy reference norms. In both plots, the big solid dots indicate the mean; smaller dots, individual data points; and error bars, SEM. ^a^*P* < .001. ^b^*P* < .05.

The NTN_A+_ subgroup showed significantly higher amygdala activity compared with the NTN_A−_ subgroup (mean difference [MD], 1.66; 95% CI, 1.00-2.32; Cohen *d*, 2.7; *P* < .001) (Figure 2A). The NTN_A+_ subgroup demonstrated significantly more implicit threat bias, with slower reactions times in the implicit recognition of facial emotion test when primed with anger compared with neutral faces (MD, −0.93; 95% CI, −1.61 to 0.26; Cohen *d*, −1.48; *P* = .01) (Figure 2B). Further, we confirmed that differences between the NTN_A+_ and NTN_A−_ subgroups were not due to baseline demographics, overall symptom severity, or early life trauma assessed in the study (Table). However, there was a relatively higher distribution of PTSD symptoms and early life traumatic events in the NTN_A+_ subgroup (eFigure 2 in Supplement 2).

### Distinct Acute MDMA-Induced Neural Responses

**Figure 3.**
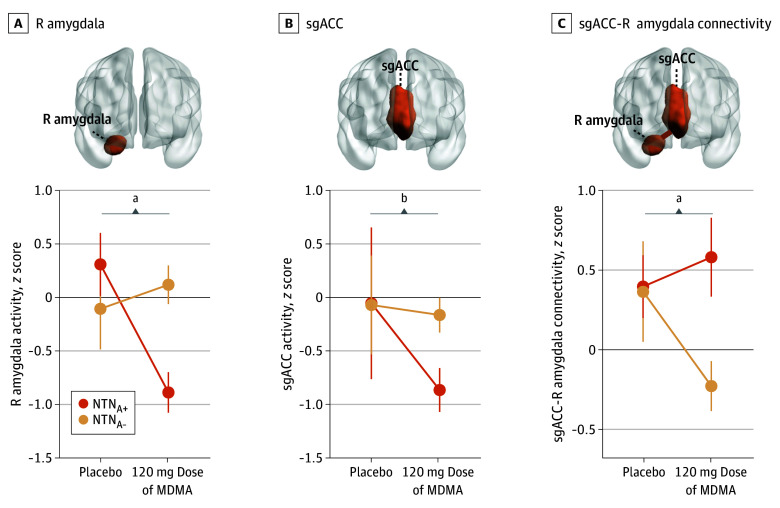
Baseline High (NTN_A+_) and Low (NTN_A−_) Negative Affect Circuit Activity Subgroups With Different Acute Neural Response to 3,4-Methylenedioxymethamphetamine (MDMA) After acute administration of MDMA at a dose of 120 mg vs placebo, the NTN_A+_ subgroup showed a greater activity reduction in the negative affect circuit in the right (R) amygdala (A) and the subgenual anterior cingulate cortex (sgACC [B]) and a greater increase in the circuit connectivity between sgACC and R amygdala (C), compared with the NTN_A−_ subgroup. In all plots, solid dots indicate the mean; error bars, SEM. ^a^*P* < .05. ^b^*P* < .01.

When examining MDMA-induced acute changes in activity and connectivity evoked by the same nonconscious threat, distinct differences were observed between the NTN_A+_ and NTN_A−_ subgroups for the 120-mg MDMA dose vs placebo. Specifically, compared with the NTN_A−_ subgroup, the NTN_A+_ subgroup exhibited a greater activity reduction induced by the 120-mg MDMA dose in the negative affect circuit in the right amygdala (contrast estimate [CE], −1.43; 95% CI, −2.60 to −0.27; Cohen *d*, −1.22; *P* = .02) (Figure 3A and eFigure 3 in Supplement 2; see eTable 1 in Supplement 2 for the model summary of the right amygdala and eTable 2 in Supplement 2 for the model summary of the left amygdala) and in the sgACC (CE, −1.48; 95% CI, −2.42 to −0.54; Cohen *d*, −1.56; *P* < .01) (Figure 3B and eFigure 3 and eTable 3 in Supplement 2), and a greater increase in the circuit connectivity between the sgACC and the right amygdala (CE, 0.65; 95% CI, 0.02-1.28; Cohen *d*, 1.02; *P* = .04) (Figure 3C and eFigure 3 and eTable 4 in Supplement 2).

### Different Acute MDMA-Induced Likability of Threat Expressions

When examining the effects of MDMA on implicit threat bias, we observed no significant difference between the 2 subgroups (CE, 0.45; 95% CI, −0.49 to 1.39; Cohen *d*, 0.38; *P* = .34) (eTable 5 in Supplement 2). However, when examining the effects of MDMA on the perceived likability of the same threat facial expressions, distinct differences were observed between the NTN_A+_ and NTN_A−_ subgroups for the 120-mg MDMA condition compared with placebo. Specifically, compared with the NTN_A−_ subgroup, the NTN_A+_ subgroup demonstrated a significant increase in likability ratings for threat faces—particularly angry faces—under the 120-mg MDMA dose (CE, 14.38; 95% CI, 1.46 to 27.29; Cohen *d*, 0.86; *P* = .03) (eFigure 3 and eTable 6 in Supplement 2).

### Divergent Acute MDMA-Induced Affective Responses

In addition to the different neural and behavioral profiles observed between the NTN_A+_ and NTN_A−_ subgroups, self-reported affective response also differed for the 120-mg MDMA dose vs placebo. Compared with the NTN_A−_ subgroup, the NTN_A+_ subgroup reported less increase in the positively valenced experiences of wanting to be with others (CE, −25.00; 95% CI, −48.14 to −1.86; Cohen *d*, −0.84; *P* = .04) (eFigure 3 and eTable 7 in Supplement 2) and feeling secure (CE, −20.00; 95% CI, −38.90 to −1.10; Cohen *d*, −0.82; *P* = .04) (eFigure 3 and eTable 8 in Supplement 2). The attenuated increase in wanting to be with others in the NTN_A+_ compared with the NTN_A−_ subgroup was also observed in the 80-mg MDMA dose vs placebo (CE, −26.87; 95% CI, −50.97 to −2.78; Cohen *d*, −0.86; *P* = .03) (eFigure 3 and eTable 7 in Supplement 2). In contrast, for the 120-mg MDMA dose vs placebo, compared with the NTN_A−_ subgroup, the NTN_A+_ subgroup reported a greater increase in the negatively valenced experiences of anxiety (CE, 8.44; 95% CI, 1.38-15.49; Cohen *d*, 0.93; *P* = .02) (eFigure 3 and eTable 9 in Supplement 2) but not impaired control and cognition (CE, 11.07; 95% CI, −0.53 to 22.67; Cohen *d*, 0.74; *P* = .06) (eTable 10 in Supplement 2).

Our results remained consistent when using multiple imputation to address missing values (eMethods, eResults, and eTables 11-13 in Supplement 2). These quantitative measures were consistent with the recorded narratives (eTable 14 in Supplement 2), in which the NTN_A+_ subgroup reported introspective and emotionally nuanced experiences during the 120-mg MDMA administration, while the NTN_A−_ subgroup described more positive and euphoric experiences.

### Masking Analysis

Across all conditions, dose conditions were correctly identified for 39 of 46 visits (85%) for study clinicians, 38 of 47 (81%) for research coordinators, and 34 of 44 (77%) for participants. When analyzing each dose condition separately, the highest accuracy was observed for the placebo condition, with lower accuracies for the 80-mg and 120-mg MDMA conditions (eTable 15 in Supplement 2).

## Discussion

Our findings in this randomized clinical trial demonstrate the importance of stratifying individuals by baseline circuit activity to differentiate acute MDMA-induced neural, behavioral, and affective responses. Specifically, following acute administration of 120 mg of MDMA, participants in the NTN_A+_ subgroup exhibited significant reductions in amygdala and sgACC activity, as well as increased connectivity between these regions. These results underscore the potential of using neural circuit markers not only to stratify patients based on threat reactivity, but also to quantify the mechanism-specific responses elicited by MDMA.

Notably, the negative affect circuit markers that stratified participants and exhibited acute changes with MDMA were elicited during the nonconscious processing of threat stimuli. Implicit threats directly engage the amygdala, enabling the detecting of danger on an unconscious level, bypassing cognitive systems.^32^ The sgACC is functionally connected to the amygdala in response to implicit threats^16^ and plays a key role in the implicit regulation of amygdala activity.^33^ Heightened amygdala activity during nonconscious threat processing is a well-established marker of threat reactivity in both PTSD and MDD,^13,34^ and it has been linked to poor responses to medication and psychotherapies.^19,20^ In PTSD, successful therapeutic extinction of implicit threat conditioning has been associated with increased amygdala-sgACC connectivity,^35^ highlighting the importance of this circuit in therapeutic outcomes.

In the NTN_A+_ subgroup, the 120-mg MDMA dose alone—without accompanying therapy but within a supportive research environment—significantly reduced threat-related amygdala activity. This result aligns with prior fMRI findings in nonclinical participants^26^ but is specific to the MDMA dose of 120 mg. It reinforces the understanding of MDMA-assisted therapy, in which MDMA dampens automatic threat reactivity,^36^ enabling individuals to engage more emotionally with threat- and trauma-related memories. Our findings suggest that the pharmacological effects of MDMA play a crucial role in creating a more receptive neural state for processing trauma-related information. Additionally, the observed increase in amygdala-sgACC connectivity in the NTN_A+_ subgroup following MDMA administration indicates that these effects may enhance the implicit regulation of threat- or trauma-related stimuli. This implicit regulation facilitates contextual processing, potentially enabling participants to shift from hyperengagement with traumatic material to more integrated and contextually grounded processing.^37^ While our study used a supportive environment without any structured trauma therapy, pairing MDMA with therapy may further enable reappraisal of traumatic material within a controlled clinical setting.

Participants in the NTN_A+_ subgroup who demonstrated high baseline amygdala activity also exhibited a bias toward implicit priming by the same threat stimuli used during fMRI. This bias was reflected in slowed reaction times to implicit priming, suggesting a heightened reactivity toward threat detection. These findings indicate that implicit priming serves as a behavioral surrogate for circuit-based markers activated by implicit threat stimuli, making it useful to complement fMRI measures for stratifying participants.

Building on the neural findings, the NTN_A+_ subgroup also showed an increase in the likability ratings of threat faces under the 120-mg dose of MDMA. This suggests MDMA’s potential to soften the emotional impact of threat-related stimuli in individuals with heightened baseline negative affect circuit activity. However, the NTN_A+_ subgroup reported subjective feelings of increased anxiety, despite the reduction in both amygdala activity and increased likeability for threat stimuli. These subjective feelings could reflect the discomfort of engaging with threat-related material, consistent with previous observations.^38^

Stratification in our study offers a promising approach for future US Food and Drug Administration (FDA) trials by identifying responders and nonresponders prior to treatment, enabling more targeted participant selection. This strategy can streamline trial designs, improve efficiency, and accelerate the development of MDMA-based therapies. Moreover, it mitigates potential risks, such as MDMA-induced vulnerability or misuse, ensuring safer and more effective clinical applications.

Our study provides insights into the standalone pharmacological effects of MDMA, aligning with the FDA’s call to better understand MDMA independent of therapy.^39^ By including a true baseline visit, we accurately measured predrug neural activity, ensuring that effects observed between the NTN_A+_ and NTN_A−_ subgroups were not conflated with placebo responses. The addition of an 80-mg MDMA condition alongside the 120-mg dose reduced functional unmasking by lowering participants’ ability to identify treatment conditions, with accuracy notably lower than in MDMA-assisted therapy trials,^23^ particularly for individuals in the NTN_A−_ subgroup, in which accuracy ranged from 50% to 60%.

### Limitations

While our study provides valuable insights, it is important to acknowledge several limitations. The small sample size limits generalizability, and validation in larger cohorts is needed. Since the study was conducted in nonclinical participants, replication in clinical samples is essential to determine whether individuals with NTN_A+_ derive greater benefit from MDMA-based therapies. Although baseline clinical measures did not show significant differences between subgroups, trends in PTSD symptoms and early life trauma within the NTN_A+_ subgroup warrant further investigation in clinical settings. Furthermore, future research should explore additional neural pathways beyond the amygdala and negative affect circuit to gain a more comprehensive understanding of MDMA’s mechanisms of action.

## Conclusions

In this randomized clinical trial of MDMA’s acute profiles, baseline stratification emerged as a crucial factor in estimating MDMA-induced neural, behavioral, and affective responses. The study highlighted that neuroimaging-based stratification effectively captured individual variability among participants with varying PTSD symptoms and early life trauma histories. These findings offer preliminary evidence supporting the use of neuroimaging to tailor MDMA-based treatments and enhance therapeutic outcomes.

## Notes

Supplement 1.
Trial Protocol

Supplement 2. **eMethods.** Prior Power Analysis, Participants, and Assessments **eResults.** Multiple Imputation Analysis for Imaging Data **eTable 1.** Summary of the Linear Mixed Model for Right Amygdala Activity **eTable 2.** Summary of the Linear Mixed Model for Left Amygdala Activity **eTable 3.** Summary of the Linear Mixed Model for sgACC Activity **eTable 4.** Summary of the Linear Mixed Model for sgACC to Right Amygdala Connectivity **eTable 5.** Summary of the Linear Mixed Model for Implicit Threat Bias **eTable 6.** Summary of the Linear Mixed Model for Threat Face Likability **eTable 7.** Summary of the Linear Mixed Model for VAS Wanting to Be With Other People **eTable 8.** Summary of the Linear Mixed Model for VAS Feeling Secure **eTable 9.** Summary of the Linear Mixed Model for Anxiety **eTable 10.** Summary of the Linear Mixed Model for Impaired Control and Cognition **eTable 11.** Summary of the Linear Mixed Model for Right Amygdala Activity With Multiple Imputation **eTable 12.** Summary of the Linear Mixed Model for sgACC Activity With Multiple Imputation **eTable 13.** Summary of the Linear Mixed Model for sgACC to Right Amygdala Connectivity With Multiple Imputation **eTable 14.** Summary of Participants’ Neural, Behavioral, and Affective Changes and Quotations After Administration of 120 mg of MDMA vs Placebo **eTable 15.** Accuracy of Dose Identification Across Different Drug Conditions **eFigure 1.** Study Design and Baseline Stratification for the Randomized Controlled Mechanistic MDMA Trial **eFigure 2.** Baseline Clinical Symptoms for High (NTN_A+_) and Low (NTN_A−_) Negative Affect Circuit Activity Subgroups Evoked by Nonconscious Threat **eFigure 3.** Acute Neural, Behavioral, and Affective Response to Placebo and MDMA at 80 mg and 120 mg for High (NTN_A+_) and Low (NTN_A−_) Negative Affect Circuit Activity Subgroups Evoked by Nonconscious Threat
eReferences.

Supplement 3.
Data Sharing Statement
