# Supplementary material for: Negative Affect Circuit Subtypes and Neural, Behavioral, and Affective Responses to MDMA: A Randomized Clinical Trial
Source: JAMA Netw Open. 2025 Apr 30;8(4):e257803. doi: 10.1001/jamanetworkopen.2025.7803 (PMC12044494; doi:10.1001/jamanetworkopen.2025.7803)
Supplement: Supplement 1. — Trial Protocol [file jamanetwopen-e257803-s001.pdf]

Stanford Regulating Circuits of the Brain Study- MDMA (RBRAIN-MDMA): study protocol for a double-blinded, within-subjects, placebo-and-baseline-controlled randomized mechanistic trial of acute MDMA administration

PI: Leanne Williams
Department of Psychiatry and Behavioral Sciences, Stanford University, Stanford, CA, USA; Sierra-Pacific Mental Illness Research, Education, and Clinical Center (MIRECC) Veterans Affairs Palo Alto Health Care System, Palo Alto, CA, USA

Trial registration: Stanford Regulating Circuits of the Brain Study (RBRAIN) - **ClinicalTrials.gov Identifier:** NCT04060108
URL: https://clinicaltrials.gov/study/NCT04060108
This work is supported by the National Institute on Drug Abuse [grant number P50DA042012].

1. Objectives

1.1 Primary objective: To characterize in depth the acute neural, behavioral, and experiential profile induced by MDMA during conscious and nonconscious processing of positive and negative emotions, and to examine how these profiles vary as a function of baseline stratification based on circuit profiles.

1.2 Secondary objective: To characterize in depth the acute neural, behavioral, and experiential profile induced by MDMA during cognitive processing, monetary reward processing, and naturalistic resting-state, and to examine how this profile varies as a function of baseline stratification based on circuit profiles.

2. Methods

2.1 Trial design and overview

A mechanistic clinical MDMA trial to investigate the effects of MDMA on human brain circuit function using a double-blinded, within-subjects, placebo-and-baseline-controlled randomized design. The design includes four testing sessions including baseline, placebo, 80mg MDMA hydrochloride (HCl), and 120mg MDMA HCl, randomized within subjects.

2.2 Study setting and personnel

Data will be collected at Stanford University in Stanford, CA. Eligibility surveys will be collected through REDCap in the participant's native environment. Screening visits will be conducted at the Stanford Clinical and Translational Research Unit (CTRU), a research clinic located on the Stanford Campus. Baseline and drug visits will be conducted at the Stanford Center for Cognitive and Neurobiological Imaging (CNI), a human magnetic resonance imaging (MRI) facility dedicated to research.

In-person screening procedures will be conducted by trained medical professionals including phlebotomists (blood draw), research nurses (ECG and vital signs), trained research coordinators (drug and psychiatric histories), and licensed study clinicians (physical examinations). Baseline and drug visit procedures will be conducted by trained research coordinators (study assessments and scanning) and licensed study clinicians (drug administration, monitoring, and safety assessments). These procedures are not part of standard care or intended as an intervention.

2.3 Informed consent

Informed consent procedures will be conducted by a trained research coordinator during screening visits. The research coordinator thoroughly explains the study protocol and answers the participant's questions before consent is obtained.

2.4 Additional consent provisions for collection and use of participant data and biological specimens

During consent, study participants are informed that information from analyses of their coded samples and their coded medical information will be put into one of the National Institutes of Health (NIH) databases, along with information from the other research participants, and will be used for future research. Saliva samples are collected at multiple timepoints during drug visits for hormone analysis. A participant may opt-out of saliva collection for analysis during consent. The decision to withhold consent for saliva sampling collection will not determine a participant's eligibility to be in the study.

2.5 Interventions

2.5.1 Intervention description

After baseline visit, each participant will receive placebo, 80mg MDMA HCl, and 120mg MDMA HCl in a randomized order. Given the within-subjects design of the study, each participant will receive all three of the specified doses across the duration of the trial. Drug visits will be scheduled 10-14 days apart to avoid drug carry-over effects. Participants will arrive fasted for drug visits in the morning to reduce the risk of emesis. They will complete a urine drug screen and pregnancy test (if applicable) and have their baseline vitals recorded at the Stanford Center for Cognitive and Neurobiological Imaging, where they will be met by the study clinician. Participants will be instructed to orally ingest pills with water or an electrolyte solution. For safety reasons, pulse, blood pressure, temperature, and oxygen saturation will be monitored and recorded every 30 minutes throughout the drug visit. The total visit time for drug visits will be 6-8 hours.

2.6 Randomization and blinding

2.6.1 Sequence generation

Participants will be randomly assigned a dose order (80mg MDMA HCl, 120mg MDMA HCl, and placebo), and each participant will receive all three doses.

2.6.2 Concealment mechanism

With our analysis plan and recruitment goal informing the number of randomization assignments, we will use randomization.com to create a random order dose assignment (80mg, 120mg, and placebo) for each of the 40 study IDs. Study IDs are sequential, starting with 001 and ending with 040.

2.6.3 Implementation

Unblinded study personnel are responsible for generating the random dose assignments. None of the blinded study staff had access to the subject ID dose assignments at any point in the randomization process. Participants are considered to be enrolled in the study once they have finished the in-person screening visit and met all the criteria. After successful enrollment, the participant is assigned a study ID, with the first participant enrolled assigned 001, the second 002 and so on. MDMA and placebo pills were obtained from the Multidisciplinary Association for Psychedelic Studies (MAPS). Before a study visit

involving drug administration, the unblinded study personnel will reference the subject ID and the visit number on the randomization assignment document to make the dose determination. For example, if subject 001 is scheduled for their second drug visit, the unblinded personnel would reference the randomization assignment document for 001, look for their assigned dose for the second drug visit, and count pills according to the information on the assignment. The drug will be delivered to the blinded research team with a label stating the subject ID and visit number.

2.6.4 Blinded personnel

The study design is double blind, both the participant and the research team, including study clinicians, are blinded to the dose assignment. Blinded research staff includes clinical research coordinators, study clinicians, research nurses, and scanning personnel. All data collection is conducted by the blinded research team. Data analysts for both the interim analysis and the final analysis are unblinded.

2.6.5 Procedure for unblinding if needed

Situations in which the study team may need to break the blind may include an allergic reaction to the study drug or a psychological response to the drug that, in the investigator's opinion, requires unblinding to properly assess. The decision to unblind the study staff will be determined by the clinician present at drug visits if unblinding must occur outside of a study visit for any reason.
There are two unblinded individuals who have access to the randomization assignment, chosen at the beginning of the study and not involved in data collection. In the event the research staff must be unblinded, the contact information for both individuals (phone, email) is available to all blinded research staff. Study staff will communicate the study ID and the unblinded staff will respond with the complete randomization assignment for that participant, all three drug visits.

2.7 Emergency procedure and criteria for discontinuing or modifying allocated interventions

With input from medical professionals, the following procedures were drafted to address possible emergency scenarios during participant drug sessions:
Myocardial Infarction

- Administer 162 mg of aspirin (chewable).
- Give sublingual nitroglycerin (0.4mg) unless systolic arterial pressure < 90 mmHg or heart rate is < 50 bpm or > 100 bpm.

Oxygen Desaturation
Oxygen desaturation is considered a saturation of less than 85%.

- Administer nasal cannula oxygen at 1-6 L/min, titrating to maintain oxygen saturation above 90%.
- If, in 3 minutes, saturation levels do not reach 90%, call 911.

- If participants spontaneously report subjective feelings of air hunger with no evidence of O2 desaturation, they will be given the option of supplemental O2 by nasal cannula.

Severe Hypertension

Severely elevated blood pressure will be considered a systolic blood pressure > 200 or a diastolic blood pressure > 115 mmHg.

- Attempt non-pharmacologic interventions, such as dimming the lights and creating a relaxing environment.
- Take three consecutive blood pressure measurements, 5 minutes apart, with ECG monitoring. If blood pressure does not decrease after three measurements, the research visit will be discontinued.
- If there is no decrease after the 3rd measurement OR the participant reports chest pain at any time in conjunction with high blood pressure OR has ECG changes suggestive of ischemia in conjunction with high blood pressure, the study clinician will administer NitroStat 0.4 mg sublingual tablet AND call 911.
- If the participant experiences symptoms such as blurred vision, headache, or chest pain in conjunction with severely elevated blood pressure, 911 will be called immediately.

Bradycardia/tachycardia

- Bradycardia is defined as a heart rate < 50 bpm, except in individuals with a known baseline heart rate below this threshold and high exertional capacity. Tachycardia is defined as a heart rate > 150 bpm.
- Three consecutive heart rate measurements will be taken 5 minutes apart, along with an ECG reading. If the heart rate does not start increasing (for bradycardia) or decreasing (for tachycardia) after three measurements, the research visit will be discontinued.
- If the heart rate is < 50 bpm for three consecutive measurements and the participant shows signs of poor perfusion (e.g., altered mental status, signs of shock, chest pain), atropine 0.4mg will be administered AND call 911.
- If the heart rate is > 150 bpm and the participant is stable but the rhythm is irregular on ECG, call 911.
- Determine if a pulse is present:
    - If no pulse (e.g., ventricular tachycardia, ventricular fibrillation, or pulseless electrical activity): Immediately initiate basic life support (BLS) and advanced life support (ACLS) protocol for cardiac arrest (see below).
    - If a pulse is present: Provide oxygen if hypoxic AND call 911 AND insert IV if time while awaiting emergency response.

- Note: We do not have the ability to perform synchronized cardioversion or administer antiarrhythmics.

Cardiac Arrest

- Research visit will be discontinued.
- The clinical research coordinator will call 911, while the study clinician initiates BLS and ACLS protocols. Specifically, CPR will be administered and AED pads placed by an ACLS/BLS certified provider. Shock will be administered by an ACLS/BLS certified individual according to the directions of the AED (located in the scan room within CNI) with continued CPR and concurrent administration of epinephrine 1 mg IV every 3-5 minutes until the ambulance arrives.
    - Epinephrine will need to be diluted with 9 mL of normal saline to create a 1:10,000 (1mg/10mL) concentration.
        - Start with a 10 mL normal saline flush
        - Waste 1 mL from the flush
        - Draw 1 mL of epinephrine (1mg/mL) into the flush and mix. Remove any air bubbles prior to IV administration.
        - DO NOT mix ahead of time

Allergic Reaction

- Research visit will be discontinued.
- In case of mild to moderate allergic reaction, diphenhydramine 25mg PO will be administered by the clinician. Mild symptoms could include though are not limited to hives/rashes and itching.
- If symptoms are severe, including but not limited to swelling of the throat/and or tongue, difficulty breathing or swallowing, and/or drop in blood pressure, administer EpiPen 0.3mg Auto-injector, preferably by the clinician. 911 should be called simultaneously by a clinical research coordinator.
- For oxygen saturation <85%, a nasal cannula will be placed by the clinician until saturation reaches at least 90%.

Nausea

If the subject reports that they are feeling nauseous, the following procedure will be followed:

- The clinician will ask the subject if their nausea is mild, moderate, or severe.
- If the subject reports that their nausea is Moderate or Severe, the study clinician will administer 4mg of Zofran, sublingual.
- If the subject reports that their nausea persists and that it is severe, 911 will be called.
- All Other Emergencies
    - 911 will be called. The Palo Alto Fire Department response time from the time of the call to the scanning facility is 6 minutes or less, 90% of the time.

2.8 Relevant concomitant care permitted or prohibited during the trial

Research coordinators will record all concomitant medications participants use regularly or intermittently beginning at the in-person screening visit and at each consecutive visit throughout the study. All psychoactive medications, herbal supplements, non-prescription medications, and prescription medications must be reviewed by the research team prior to a participant being enrolled in the study. Emergency medications including nitroglycerin tablets, epinephrine auto-injector, diphenhydramine capsules, atropine sulfate, epinephrine ampoules, diphenhydramine vials, and sublingual Zofran will be kept onsite for administration by the study clinician in the case of emergency or participant need.

2.9 Eligibility criteria

Inclusion Criteria:

- Ages 18-55
- All genders and ethno-racial categories
- At least 2+ prior uses of MDMA when aged 18 years or older and have reported no serious adverse reactions from MDMA or ecstasy
- Able to swallow capsules
- Able to receive an MRI
- Able to comply with study procedures
- Able and willing to enroll and provide written informed consent

Exclusion Criteria:

- Current mood or anxiety disorder
- Current or past eating, bipolar, or psychotic disorder
- Current substance or alcohol use disorder
- Current suicide risk as assessed by psychiatric interview and clinical judgement of the investigator
- Schizophrenia in a first degree relative
- Allergy or hypersensitivity to MDMA
- Have used Ecstasy/MDMA within 6 months of the first study dose; or have previously participated in a MAPS-sponsored MDMA clinical trial
- Concurrent use of any medication or substance that might increase the risk of participation and/or interact with MDMA
- Current use of any psychotropic medication (a wash-out period of 5 half-lives will be required prior to drug visits followed by a 1-week stabilization period, if the participant reports recently discontinuing a psychotropic medication)

- Unable or unwilling to agree to refrain from using any psychoactive substances, supplements, and nonprescription medications starting 1-week prior to study start and for duration of study
- Unable or unwilling to refrain from using caffeine for 12 hours before and 10 hours after drug administration
- Direct physical access to or routine handling of addicting drugs in the regular course of work duties
- Current use of any opioids, including codeine, hydrocodone, and morphine
- Positive test on urine drug screen for illicit and/or drugs of abuse at screening and prior to study drug administration
- Body Mass Index (BMI) outside of healthy range (18-30)
- Pregnant or nursing
- Renal/hepatic impairment (assessed via laboratory tests during initial screening appointment)
- Uncontrolled hypertension as defined by a systolic blood pressure >/=140 mmHg or diastolic blood pressure >/= 90 mmHg on two of three measurements at least 15 minutes apart at initial screening appointment
- Heart rate <50bpm or >150bpm assessed at initial screening visit
- Current chronic congestive heart failure, tachyarrhythmias, myocardial ischemia (assessed via Electrocardiogram (ECG) at initial screening appointment)
- Marked Baseline prolongation of QT/QTc interval e.g., repeated demonstration of a QTc interval >450 milliseconds (ms) in males and >460 ms in females. For transgender or non-binary participants, QTc interval will be evaluated based on sex assigned at birth, unless the participant has been on hormonal treatment for 5 or more years
- Current or history of significant (controlled or uncontrolled) hematological, endocrine, cerebrovascular, cardiovascular, coronary, pulmonary, renal, gastrointestinal, immunocompromising, or neurological disease, including seizure disorder, or any other medical disorder judged by the investigator to significantly increase the risk of MDMA administration (participants with hypothyroidism who are on adequate and stable thyroid replacement will not be excluded).
- History of additional risk factors for Torsade de pointes (e.g., heart failure, hypokalemia, family history of Long QT Syndrome)
- History of hyponatremia or hyperthermia
- Inability to speak, read or understand English at a 5th grade level or severe hearing impairment
- Plan to move out of the area during the study period
- An exclusionary metal device as determined by the discretion of the Clinical Investigator.

2.10 Recruitment and Study Cohort

Healthy volunteers who report ≥ 2 uses of MDMA when they were 18 years or older, recruited to attain 4 sessions of data during functional brain imaging. A total of 40 participants will be recruited, equal

numbers of males and females will be recruited. Participants will be recruited through Facebook Ads using IRB-approved material. Individuals who express interest in the study will be directed to an online screening survey in REDCap. Completed screening surveys will be reviewed by research coordinators trained on the study protocol. Surveys will collect information about demographics, psychiatric diagnoses, substance use, and MRI compatibility. Individuals that are eligible by the criteria included in the screening survey will be contacted by a research coordinator to schedule a phone call. On this phone call, research coordinators will give the individuals additional information about the study and collect additional demographic information. Eligible individuals at this point will be consented by a research coordinator and will schedule an in-person screening visit at a research clinic to fully determine eligibility. The following procedures and assessments will be performed at the screening visit: ECG, physical examination, blood draw, vital signs, weight, urine drug test, pregnancy test (if applicable), Hamilton Anxiety Rating Scale (HAM-A), Hamilton Depression Rating Scale (HAM-D)[1,2], the Mini International Neuropsychiatric Interview (MINI)[3].

2.11 Data collection and management

2.11.1 Plans for assessment and collection of outcomes

Outcomes for the study will be measured via functional neuroimaging data, self-report questionnaires, and behavioral measures. Neuroimaging data will be collected at baseline and each of the drug visits. Self-report questionnaires will be collected at baseline, each of three drug visits, and at 1-week, and 1-month follow ups. Behavioral measures will be collected at baseline and each of three drug visits. Self-report questionnaires will be collected via REDCap. The only exceptions to this method of administration are the Visual Analog Scale (VAS), the Brief Psychiatric Rating Scale (BPRS), and the Clinician-Administered Dissociative States Scale (CADSS), which will be administered verbally by a trained clinician (BPRS and CADSS) or research coordinator (VAS). Collected measures and their descriptions are included below.

2.11.2 Functional Neuroimaging Data

2.11.2.1 Baseline and Drug Visit scans

Resting State: Participants are instructed to stare at a white cross on a black background. During this time, their eyes will be monitored using an eye-tracker by the study coordinator to ensure that the participant is not asleep. Two separate 5-minute and 12-second runs of resting state data will be acquired, one with a posterior-anterior phase encoding direction and the other with a posterior-anterior direction.

Monetary Incentive Delay (MID): Participants watch the screen and see a cue in the form of a circle or square, either of which can be empty or dashed, wait a delay of a varied amount of time, and then seen the target (a white triangle), which they are instructed to respond to with a button press as quickly as possible. The participant is instructed to attempt to press a button while the target is on the screen to

either win money or avoid losing money. A feedback slide is presented after each trial to inform the participant whether they are successful on that trial, and the resulting gain or loss of money. The task is ten minutes long: 2(valence) x2(magnitude)) = 4 conditions x 12 trials/condition x 12 sec avg/trial (incl./4 sec avg interval). This task is meant to elicit neural responses to monetary gain (non-social reward), monetary loss (punishment), and no monetary outcome (control) in striatal and medial forebrain circuitry[4].

Facial Expressions of Emotion Task (FEET, conscious and nonconscious): Participants are presented with two 5-minute and 8-second runs of 3D evoked facial expression stimuli in pseudorandom order – one consciously perceived and one nonconsciously perceived. Each run consists of five repeated blocks of eight stimuli per block, for sad, threat evoked by fear, threat evoked by anger, and happy, relative to the neutral block. Participants are instructed to actively attend to answer post-scan questions about these faces, and we control for active attention by monitoring alertness with an eye tracking system. In the conscious run, stimuli are presented for 500ms with a 750ms interstimulus interval. In the nonconscious run, the same stimuli are presented using a backward-masking design to prevent awareness: each face is shown for 16.7ms, immediately followed by a neutral face mask for 150.3ms, with a 1083ms interstimulus interval to match that of the conscious condition. To control for perceptual priming from the difference in fear-neutral versus neutral-neutral pairs, the neutral faces are spatially offset by one degree in random directions[5].

Go-NoGo: Participants are presented with 240 trials in pseudorandom order over a 5-minute and 8-second run, consisting of 180 'Go' trials and 60 'NoGo' trials. On 'Go' trials, the word "press" is displayed in green, and participants are instructed to respond as quickly as possible. On 'NoGo' trials, the word "press" appears in red, and participants are instructed to withhold their response. Each stimulus is presented for 500ms, followed by a 750ms interstimulus interval[5].

2.11.3 Self-Report Questionnaires

2.11.3.1 Baseline Visit

How Are You Today

A 11-item questionnaire created for the study asking patients about recent sleep, food and drug intake, and how the participant feels the day of assessment.

Alcohol Use Disorders Identification Test (AUDIT)

A 10-item screening questionnaire to assess hazardous and harmful alcohol consumption[6].

Beck Depression Inventory-II (BDI-II)

The BDI-II is a revision of the BDI, a 21-item self-report measure that measures depression symptom severity[7].

Beck Anxiety Inventory (BAI)

The BAI is a 21-item self-report questionnaire that assesses the severity of anxiety symptoms[8].

Behavioral Inhibition Scale (BIS)/Behavioral Activation Scale (BAS)

A 24-item self-report questionnaire designed to measure two motivational systems: the behavioral inhibition system (BIS), which corresponds to motivation to avoid aversive outcomes, and the behavioral activation system (BAS), which corresponds to motivation to approach goal-oriented outcomes[9].

Cannabis Use Disorder Identification Test (CUDIT)

An 8-item, self-report screening instrument which assesses cannabis abuse over the past 6 months[10].

Barratt Impulsiveness Scale (BIS-11)

The BIS-11 is a 30-item self-report questionnaire designed to assess the personality and behavioral construct of impulsiveness across multiple domains[11].

Edinburgh Handedness Inventory (EHI)

A 10-item self-response inventory that determines handedness dominance in everyday activities[12].

Satisfaction With Life Scale (SWLS)

A 5-item instrument designed to measure global cognitive judgments of satisfaction with one's life[13].

Brief COPE (COPE)

A 28-item multidimensional measure of strategies used for coping or regulating cognitions in response to stressors[14].

Depression Anxiety Stress Scales (21-item DASS)

A 21-item self-report questionnaire designed to assess the negative emotional states on three subscales, depression, anxiety, and stress over a timescale of a week[15].

Early Life Stress Questionnaire (ELSQ)

The ELSQ is a self-report measure used to assess exposure to stressful or traumatic events during childhood and adolescence[16].

Emotion Regulation Questionnaire (ERQ)

A 10-item self-report scale designed to assess habitual use of two commonly used strategies to alter emotion: cognitive reappraisal and expressive suppression[17].

Generalized Anxiety Disorder Questionnaire 7-item (GAD-7)

A 7-item self-reported questionnaire for screening and severity measuring of generalized anxiety disorder (GAD)[18].

Health Productivity Questionnaire (HPQ)

A self-report instrument designed to estimate the workplace costs of health problems in terms of reduced job performance, sickness absence, and work-related accidents-injuries[19].

World Health Organization Quality of Life Scale (WHOQOL-BREF)

The WHOQOL-BREF is a 26-item self-report instrument that assesses quality of life across four domains: physical health, psychological well-being, social relationships, and environment[20].

Fagerstrom Nicotine Dependence Scale (FNDS)

A 6-item questionnaire that evaluates the quantity of cigarette consumption, the compulsion to use, and dependence[21].

Mood Anxiety Symptom Questionnaire (MASQ)

A 30-item questionnaire based on the Tripartite Model of Anxiety and Mood that provides scores in three domains: General Distress, Anhedonic Depression, and Anxious Arousal[22].

Positive and Negative Affect Schedule (PANAS)

A questionnaire with a total of 20 questions, with 10 items measuring positive affect and 10 items measuring negative affect[23].

Post-Traumatic Stress Disorder Checklist-Civilian (PCL-C)

A standardized self-report rating scale for PTSD comprising 17 items that correspond to the key symptoms of PTSD[24].

Patient Health Questionnaire 9-item (PHQ-9)

A self-administered version of the PRIME-MD diagnostic instrument for common mental disorders. The PHQ-9 is the depression module which scores each of the 9 DSM-IV criteria[25].

Penn State Worry Questionnaire (PSWQ-10)

An adapted version of the Penn State Worry Questionnaire to assess pathological worry[26].

Quick Inventory of Depressive Symptoms- Self Report (QIDSSR)

A 16-item depression scale, yielding scores from 0-27, which covers the symptom domains of major depressive disorder, for the time frame of the past week[27].

NIDA Modified Alcohol, Smoking, and Substance Involvement Screening Test (NMASSIST)

A modified version of the World Health Organization's Alcohol, Smoking, and Substance Involvement Screening Test (ASSIST) that provides information about the substances people have ever used in their lifetime, the substances used in the last three months, problems related to substance use, risk of current or future harm, dependence, and injecting drug use[28].

Ruminative Response Styles Scale (RRS)

A 22-item scale that measures two aspects of rumination, brooding and reflective pondering[29].

Healthy Days Measures (HRQOL-4)

A 4-item measure of health-related quality of life[30].

Post-traumatic Stress Disorder Checklist for DSM-5 (PCL5)

A 20-item measure that assesses the 20 DSM-5 symptoms of PTSD[31].

Interpersonal Reactivity Index (IRI)

A measure of dispositional empathy that takes as its starting point the notion that empathy consists of a set of separate but related constructs. The instrument contains four seven-item subscales, each tapping a separate facet of empathy[32].

Big Five Inventory (BFI)

An instrument designed to assess the personality of individuals[33].

Social Functioning and Adjustment Scale (SOFAS)

Measures overall social and occupational functioning on a scale of 0–100 with 100 being superior functioning in a wide range of activities and the lower limits of the scale representing an inability to maintain personal hygiene or to function without hurting self or others or without considerable support[34].

2.11.3.2 Drug Session Visit

Clinician-Administered Dissociative States Scale (CADSS)

A 23-item self-report questionnaire administered by a study clinician to assess present-state dissociative symptoms[35].

Rotter's Locus of Control (RLOC)

Evaluates internal and external beliefs regarding recovery from traumatic events that have been experienced[36].

5-Dimensional Altered States of Consciousness Rating Scale (5D-ASC)

A 94-item self-report questionnaire designed to probe alterations in consciousness using a sliding scale[37,38].

Visual Analog Scale (VAS)

A custom self-report questionnaire assessing the level of subjectively experienced various mood and feelings on a scale from 0-100[39].

Depression Anxiety Stress Scales (21-item DASS)

A 21-item self-report questionnaire designed to assess the negative emotional states on three subscales, depression, anxiety, and stress over a timescale of a week[40].

Brief Risk-Resilience Index for Screening (mini-BRISC)

A 15-item self-report questionnaire designed to assess emotional health and coping across three domains: negativity bias, resilience, and social capacity[41].

Snaith-Hamilton Pleasure Scale (SHAPS)

A 14-item self-report questionnaire designed to measure anhedonia[42].

Dimensional Apathy Scale (DAS)

A 24-item self-report questionnaire designed to assess apathy across three subscales: executive, emotional and behavioral, and cognitive initiation[43].

Motivation and Pleasure Scale-Self-Report (MAP-SR)

An 18-item self-report questionnaire designed to assess motivation and pleasure in negative symptoms[44].

The Dimensional Anhedonia Rating Scale (DARS)

A 17-item self-report questionnaire designed to assess desire, motivation, effort, and consummatory pleasure across hobbies, food/drink, social activities, and sensory experience[45].

Brief Psychiatric Rating Scale (BPRS)

An 18-item self-report questionnaire administered by a study clinician to screen for the presence and severity of psychiatric symptoms related to psychosis[46].

Social Reward Questionnaire (SRQ)

An instrument to assess individual differences in the value of different social rewards[47]

MID Cue Rating

A custom survey created for the study rating the likability of stimulus presented during the MID task on a scale from –5 to 5.

Face Likability Rating

A custom survey created for the study rating the likability of faces presented during the FEET task on a scale from 0 to 100.

Acceptance/Avoidance-Promoting Experiences Questionnaire (APEQ)

A self-report instrument to assess acceptance-related experience and avoidance-related experience[48].

Qualitative recording

Participants will be prompted to describe their experience with narratives recorded.

2.11.3.3 Follow-up (24-hour, 1-week, 1-month)

DARS

DASS 21-Items

PCLC

2.11.4 Behavioral Measures

2.11.4.1 Baseline and Drug Session Visit

Computerized tests of behavioral performance (WebNeuro)

These computerized tasks are executed on a provided computer by the participant during their study visit. The software runs each of the tasks and includes standardized task instructions, as well as measures of accuracy and reaction time. Psychometric properties have been established for each of these tests, including norms[49], construct validation[50], and validation against traditional neuropsychological tests tapping equivalent functions, test-retest reliability, and consistency across cultures[51].

The tasks are described below:

Explicit emotion identification: Participants identify the emotion of 96 facial expressions (neutral, happy, sad, fear, anger, or disgust).

Implicit emotion priming: 24 of the photographs from the explicit emotion identification task are randomly selected for each participant and are presented a second time, approximately 20 minutes after the explicit motion identification task. Each will be shown beside one of 24 new photographs showing a different individual but of the same sex and emotion. For each pairing, participants indicate which of the two faces they have seen in the previous task.

Continuous performance test: A series of 125 similar looking letters are presented to the participant on the computer screen. If the same letter appears twice in a row, the participant is required to press the spacebar.

Go-NoGo: The color of the word 'PRESS' is frequently presented in green (Go) and infrequently in red (NoGo). To assess inhibition, the participant is asked to inhibit keypress responses when the word 'PRESS' is red.

Verbal Interference Stroop: The participant is presented with colored words (red, yellow, green, or blue), one at a time. They are asked to identify the name of each colored word (ignoring the color of the word), then identify the color of each word (ignoring the name of the color) as quickly as possible.

Choice Reaction Time: Participants are asked to look at the computer screen as one of four target circles was illuminated in pseudorandom sequence over a series of trials. To assess processing speed, the participant is asked to click on the illuminated circle as quickly as possible following the presentation.

Digit Span: Participants are presented with a series of digits on the computer screen, then are asked to enter the digits presented. In part 1, participants are required to recall the digits in forward order. In part 2, they are required to recall them in reverse order. In each part, the number of digits in each sequence gradually increases from 3 to 7, with two sequences at each level. Max span indicates the maximum number of digits the participant recalled without error.

Maze: Participants are asked to discover the hidden path through a grid of circles (by trial and error) presented on the computer screen. This task measures how quickly the participant learned the route through the maze and their ability to remember that route, thus taps executive function abilities.

2.11.5 Additional Measures

2.11.5.1 Drug Session Visit

Steroid hormone assay via saliva collection: At 6 different time points throughout drug administration visits, the participant chews on a cotton swab for 30 seconds to collect saliva. A steroid hormone assay is conducted to collect data on cortisol, testosterone, progesterone, and estradiol levels.

Qualitative free responses: Subjects will be prompted to report their drug experience throughout the drug visit. Responses are recorded by a research coordinator.

2.11.6 Plans to promote participant retention and complete follow-up

To promote participant retention, participants agree to attend all four subsequent study visits at the time of the in-person screening visit. Follow-up measures will be completed via online survey at 1-week and 1-month, which research coordinators will review for completeness and follow up with participants by email as necessary. Participants who either elect to discontinue study procedures or are unable to continue study procedures will complete an abbreviated version of the study protocol according to the participant's desired level of participation and the safe level of participation as determined by the study clinician.

2.11.7 Data management

The following data management and quality control procedures will be implemented. All study data will be entered into computerized data files utilizing: (1) Microsoft Access for data entry on recruitment and testing schedule tracking; (2) REDCap survey software for real-time entry and verification of data from self-report instruments during assessment visits. All the data entry systems will employ automatic, real-time range, logic, and missing value checks. Data sets will be cleaned, verified, and archived, and then read into R (https://www.r-project.org/) or equivalent data analytics software, which also will be archived. One official copy of all the study data and a master data dictionary will be maintained and updated regularly by the study data analyst. All analytic and tracking database files will be stored in a secure Stanford network drive with daily backups. One copy of the backups will be saved on-site and one off-site. Separate archival databases will be permanently maintained. For the protection of human subjects' confidentiality, unique anonymous study IDs will be used for data storing, tracking, and reporting. The data files can be shared by authorized study personnel both on-site and in remote locations via a secure virtual private network. Data security will be ensured through industry-standard password protection and data encryption. Brain imaging data are evaluated at the time of acquisition for any observable abnormalities that require a clinical radiological report, to exclude neurological abnormalities. We transfer data from the scanner acquisition site to the project database via anonymized ID from a secure server (Flywheel). The imported raw scan files (called "NIfTI" files) are visually inspected by the experienced postdoctoral researcher overseen by Dr. Williams, prior to analysis. The files are run through a sophisticated quality control pipeline that checks for factors such as the extent of whole-brain coverage, the quality of motion correction, image registration, and parameter maps. Anonymized data will be kept in a structured file system that includes selected intermediate steps to permit the easy recalculation of data for specific analyses, using the virtual computing environment overseen by the Stanford Neurosciences Institute. Off-site backups of data are kept in a secure facility.

As an integral part of high-quality data management, we will implement a series of strategies that have proven effective in our previous clinical trials to achieve data completeness. These include the following: (1) careful staff selection and standardized training in drug administration-specific protocols, rapport building, motivational interviewing, and problem solving as appropriate to their study roles; (2) legally adequate, effective informed consent; (3) prudent human subjects incentives and flexible scheduling; (4) promotion of study identity; (5) ongoing monitoring of recruitment and retention; (6) up-to-date human subjects contact information and two emergency contacts; (7) diligent efforts to re-engage inactive human subjects; and (8) alternative means of obtaining measurements. To maximize retention and assure that human subjects fully understand the demands and nature of the study before they enroll, recruitment staff will, as part of a detailed informed consent process, carefully review study requirements with the subjects, explain the concept of random assignment and what each session involves.

2.11.8 Confidentiality

Information about study subjects will be kept confidential and managed according to the requirements of the Health Insurance Portability and Accountability Act of 1996 (HIPAA). Those regulations require a signed subject authorization informing the subject of the following:

- What protected health information (PHI) will be collected from subjects in this study
- Who will have access to that information and why
- Who will use or disclose that information
- The rights of a research subject to revoke their authorization for use of their PHI.

· In the event that a subject revokes authorization to collect or use PHI, the investigator, by regulation, retains the ability to use all information collected prior to the revocation of subject authorization. For subjects that have revoked authorization to collect or use PHI, attempts should be made to obtain permission to collect at least vital status (i.e., that the subject is alive) at the end of their scheduled study period.

2.11.9 Records Retention

Some data will initially be entered onto paper source documents and then entered into a secure database (REDCap) and some data will be entered directly into REDCap. The REDCap system is password protected only accessible to designated research staff. All raw data with identifying information will be stored in locked files or a secure server. To access the computer and the appropriate data files, the staff member must have the password and be given rights to access the data by the Principal Investigator. All paper data with identifying information will be stored in locked files (401 Quarry Rd). Electronic data will be uploaded onto a secure server (Stanford PHI Box). Data being analyzed will be identified by subject codes and identifying information will be removed. The protected health information that will be collected in this study will be limited to the least amount of information needed to accomplish the purpose of the study.

3. Neuroimaging scan parameters, preprocessing, and circuit quantification

3.1 Neuroimaging scan parameters for baseline and drug session visit

T1-Weighted Anatomical: TE = 3.548 ms, TR = 3 s, FA = 8, acquisition time = 8:33, field of view = 256 × 256 mm, 3D matrix size = 320 × 320 × 230, slice orientation = sagittal, angulation to AC-PC line, receiver bandwidth = 31.25 kHz, fat suppression = no, motion correction = PROMO, voxel size = 0.8 mm isotropic.

T2-Weighted Anatomical: TE = Maximum, TR = 2.5 s, FA = 90, acquisition time = 5:42, field of view = 256 × 256 mm, 3D matrix size = 320 × 320 × 216, slice orientation = sagittal, angulation to AC-PC line, receiver bandwidth = 125 kHz, fat suppression = no, motion correction = PROMO, voxel size = 0.8 mm isotropic**.**

Spin-echo field maps for multi-band fMRI: TE = Min Full, TR = 5.5 s, FA = 90, acquisition time = 18 s, field of view = 216 × 216 mm, 3D matrix size = 90 × 90 × 60, slice orientation = axial, angulation to

anterior commissure - posterior commissure (AC-PC) line, phase encoding = AP and PA, receiver bandwidth = 250 kHz, readout duration = 45.924 ms, echo spacing = 0.516 ms, voxel size = 2.4 mm isotropic.

Single-band reference for multi-band fMRI: TE = 30 ms, TR = 4.2 s, FA = 90, acquisition time = 13 s, field of view = 216 × 216 mm, 3D matrix size = 90 × 90 × 60, slice orientation = axial, angulation to AC-PC line, phase encoding = AP and PA, receiver bandwidth = 250 kHz, readout duration = 45.924 ms, echo spacing = 0.516 ms, number of volumes = 3, voxel size = 2.4 mm isotropic.

Multi-band fMRI: TE = 30 ms, TR = 0.71 s, FA = 54, acquisition time = 5:12 (Resting state x 2), field of view = 216 × 216 mm, 3D matrix size = 90 × 90 × 60, slice orientation = axial, angulation to AC-PC line, phase encoding = PA and AP, receiver bandwidth = 250 kHz, readout duration = 48.772 ms, echo spacing = 0.548 ms, multiband factor = 6, voxel size = 2.4 mm isotropic. The low multiband factor (6) and larger voxel size (2.4mm) increase the signal to noise ratio, especially in our subcortical structures of interest.

Spiral field map for single-band fMRI: TE = 4.5 ms, TR = 1.4 s, FA = 70, field of view = 222 x 222 mm, 3D matrix size = 256 x 256 x 45, slice orientation = axial, receiver bandwidth = 125 kHz, voxel size = 0.87 x 0.87 x 3 mm.

Single-band fMRI: TE = 27.50 ms, TR = 2 s, FA = 77, acquisition time = 5:08 (FEET and Go-NoGo) and 10:14 (MID), field of view = 222 × 222 mm, 3D matrix size = 74 × 74 × 45, slice orientation = axial, angulation to AC-PC line, phase encoding = PA, number of volumes = 154, voxel size = 3 mm isotropic.

3.2 Brain Imaging Preprocessing

All participants' data will be quantified using the Stanford Et Cere Image Processing System protocol[52,53]. An fMRI preprocessing pipeline will be completed using SPM8 (https://www.fil.ion.ucl.ac.uk/spm/software/spm8/) and FSL 5.0.9 (https://fsl.fmrib.ox.ac.uk/fsl) following previously established procedures[52,54], including realignment, co-registration, normalization to the standard Montréal Neurological Institute space, and smoothing with a Gaussian kernel of 8 mm full width at half maximum. A participant's data will be included if no more than 25% of time points (38 out of 151 frames) will be censored by frame-wise displacement ≥ 0.3mm or scaled signal intensity differences ≥ 10. A temporal mask will then be created for each censored volume and its subsequent volume, and will be used as nuisance regressors in the participant-level modeling. Additional quality control diagnostics include visual inspection of the raw fMRI time series for artifacts and signal dropout.

3.3 Definition of region of interest (ROI)

A priori defined ROIs will be used. In summary, an anatomical definition of subcortical nodes was combined with an automated meta-analysis approach to cortical nodes using neurosynth.org[55]. Neurosynth uniformity (previously called forward-inference) maps were used with a false detection rate (FDR) threshold of .01 for each circuit and defined our ROIs. A set of peaks associated with each circuit's

search term were then identified using AFNI’s 3dExtrema function. Because some terms yielded maps with excessively large spatial extent, a restriction was imposed that each peak have a minimum z-score of 6 and each region extend no farther than 10mm from the peak. For subcortical regions, neurosynth maps were restricted by anatomically defined boundaries from the AAL atlas[56] plus an additional anatomical boundary defining the ventral striatum from the FSL atlas[57]. The Talairach atlas was used to identify the anatomical location of the peak of each region, and visual inspection of masks confirmed or adjusted these automatically derived labels. In order to refine and maximize the quality of circuit definitions, we implemented the following steps in two healthy reference samples. In the first sample, each individual’s gray matter was identified by warping the output of FSL’s FMRIB's Automated Segmentation Tool (FAST) to the MNI template. Each ROI was limited to gray matter only using this procedure. Using the second reference sample, we excluded ROIs with less than 50% average overlap between the original ROI and gray matter. Next, in the first reference sample, ROIs were excluded if 95% of subjects had a temporal signal to noise ratio (tSNR) two standard deviations above the mean tSNR of a gray matter region with considerable signal drop out (peak coordinates 2, 46, -16, mean tSNR=47.03). To further establish the internal validity of circuit definitions, the internal consistency of functional connectivity between pairs of regions was assessed, excluding region pairs for which connectivity (both task and task-free) showed stronger associations with out-of-circuit region pairs than with within-circuit region pairs in a healthy sample.

3.4 Quantification of brain circuits of interest under task fMRI and task-free fMRI

For each task, each subject’s preprocessed data will be entered into a general linear model (GLM) using SPM8 (https://www.fil.ion.ucl.ac.uk/spm/software/spm8/). Task-evoked activation is quantified using a generalized linear model (GLM) in which task events are convolved with a canonical hemodynamic response function as implemented in SPM8. In this analysis, a 128s high pass filter is be applied to the data, and six realignment parameters are added to the design matrix as confounds. Residuals from these models are saved and used for the estimation of task-free connectivity (see below). Specific contrasts of interest are then computed for each circuit as follows: 1) negative affect circuit: sad > neutral conscious faces; 2) negative affect circuit: threat > neutral conscious faces; 3) negative affect circuit: threat > neutral nonconscious faces; 4) positive affect circuit: happy > neutral conscious faces; 5) cognitive control circuit: NoGo > Go trials. Measures of activation for each region of each circuit are obtained by extracting the average value of the contrast of interest.

To quantify task-based functional connectivity, we compute psychophysiological interactions (PPI) between pairs of regions belonging to the same circuit. For each region in each circuit (PPI seed), we calculate the first eigenvariate of that region’s time series and fit a whole-brain first-level GLM as described above, which consists of the psychological variable (task contrast of interest), physiological variable (region time course), and the interaction between psychological and physiological variables (PPI effect of interest). Then, we

compute the average PPI effect of interest in specific regions belonging to the same circuit in accordance with our hypothesized model of circuit dysfunction (PPI targets).

Task-free data are derived following an established procedure[58]. First, the residuals of the task effects are saved from the GLM analysis described above. Then, these residuals are band-pass filtered between 0.08 and 0.009 Hz using FSL and concatenated across tasks. We then calculate from these data the correlation coefficient of the timeseries of each region pair belonging to the default mode, attention, and salience circuits. Finally, these values are converted to Fisher z and used as measures of task-free functional connectivity.

Resting brain-wide connectivity will be quantified by Pearson correlations between brain regions. Localized resting state functional connectivity and activity will be quantified using previously determined parcellations[59,60], and provide a means to correct for multiple comparisons. We complement this approach with data-driven graph theoretical analysis to characterize whole-brain within-and between-circuit organization[61,62] and dynamic time-varying activity functional connectivity[63].

A unique feature of our image processing system is that quantified circuit measures are expressed in terms of standard deviation units from the mean of a healthy reference sample, and thus are interpretable for each individual. We refer to the resulting measures as “regional circuit scores”.

4. Outcomes

4.1 Primary Outcomes

Neuroimaging:

1. FEET (conscious and nonconscious) evoked functional activity and connectivity in negative and positive affect circuits

4.2 Secondary Outcomes

Neuroimaging:

1. Task-free intrinsic functional connectivity in default mode, attention, and salience circuits
2. Resting-state circuit activity and connectivity
3. MID evoked circuit activity and connectivity
4. Go-NoGo evoked activity and connectivity in the cognitive control circuit

Behavioral Measures

1. WebNeuro

Self-Report Questionnaires:

1. CADSS
2. 5D-ASC
3. 21-Item DASS

4. SHAPS
5. DAS
6. MAP-SR
7. DARS
8. BPRS
9. MID Cue Rating
10. Face Likability Rating
11. APEQ
12. VAS
13. SRQ

Additional Measures:

1. Steroid hormone assay via saliva collection
2. Qualitative free responses

5. Statistical methods

5.1 Statistical methods for primary outcomes

To assess MDMA-induced actuate changes in the regional circuit scores derived from the conscious and nonconscious FEET task, repeated measures linear mixed-effects models (LMMs) will be applied. Each outcome measure will serve as the dependent variable, with Dose (placebo, 80mg MDMA and 120mg MDMA) as a within-subjects fixed effect. Further, to assess whether baseline neural profile could predict distinct MDMA-induced acute changes, a median split approach to stratify participants into the high and low activity or connectivity groups and examine the modulation effect of the group. A separate LMM with Dose and Subgroup and their interaction as fixed effects will be implemented. We will focus on the between-subgroup difference between placebo and 120mg MDMA, as well as between placebo and 80mg MDMA. The inclusion of the 80mg dose enhances understanding of dose-dependent MDMA-induced effects and improves blinding. Covariates will include age, biological sex, and motion parameters if they are found to associate with outcomes. For our primary analysis on the pre-registered circuit measures, we will not perform multiple comparison correction as they measure overlapping constructs and do not meet strict criteria for independent measures. We acknowledge the issue of multiple measures, as the detailed set of assessments may measure overlapping constructs and may not meet strict criteria for independence. An alternative approach involves identifying measures that show significant MDMA-induced effects using a $p < 0.05$ threshold, examining the correlations among these effects, and selecting the measures with the greatest MDMA-induced effect sizes. Additionally, we propose an exploratory option of using principal component analysis (PCA) to form components from correlated measures to reduce dimensions of our data.

5.2 Statistical methods for secondary outcomes

For the secondary outcomes, similar repeated measures LMMs will be applied as the primary outcomes. Each secondary outcome measure will serve as the dependent variable. Covariates will include age, biological sex, and motion parameters if they are found to associate with outcomes. For our secondary analysis, well-established multiple comparison adjustment methods will be applied. For neuroimaging data, cluster-based correction methods will be used to control the family-wise error rate (FWER). For non-neuroimaging data, False Discovery Rate (FDR) adjustments will be applied to control for false positives. Additionally, we propose an exploratory option of using principal component analysis (PCA) to form components from correlated measures to reduce dimensions of our data.

Correlation analysis and cutting-edge mediation analysis will be used to understand the relation between MDMA induced brain changes and MDMA induced changes in behavioral and experiential measures.

5.3 Interim analyses

We plan the power of our study to be able to detect a small effect of MDMA on brain activation. However, recent studies have shown that this effect can be in the medium range in tasks similar to the ones we have adopted[66]. Therefore, we will run interim analyses to determine if our effects of interest can already be detected when we collect N = 17 participants.

5.4 Methods for additional analyses (e.g., subgroup analyses)

A further novel aspect of our project will be to examine sex as a biological variable as a potential moderator of MDMA-induced effects on experience, behavior, and brain function. In healthy humans, biological sex has been implicated in the subjective and physiological impact of MDMA, although the data on these effects is very limited. The acute psychoactive effects of MDMA have been reported as more pronounced in women than in men[67,68]. On the other hand, men report higher blood pressure effects on MDMA[69] than women. To our knowledge, sex differences in the acute impact of MDMA on human neural circuit function have not been investigated.

5.5 Methods in analysis to handle protocol non-adherence and any statistical methods to handle missing data

Expert researchers will visually inspect all the scans and exclude any that cannot be analyzed due to data acquisition errors or artifacts. FMRI scans with critical motion will also be excluded. In our mixed effects models, we will include all available data for each timepoint.

6. Data monitoring committee, its role and reporting structure

The PI, data manager, and study statistician will prepare a specific report for the Data Safety Monitoring Committee in advance of each bi-annual meeting. Specific attention will be given to data quality and timeliness, participant recruitment, accrual and retention, participant risk versus benefit, adverse events,

and other factors that can affect study outcome, including scientific developments that may have an impact on the safety of the participants or the ethics of the study. The procedures in place for monitoring adverse events as well as for site, data and statistical management will enable us to generate a timely report to the DSMB.

After each DSMB meeting, the DSMB will issue a report that summarizes:
1) All serious and unexpected adverse events (for example, inpatient hospitalizations) or other unanticipated problems that involve risk to study participants or others at either site, and whether these appeared related to the research assessment protocols. Reports will not specifically disclose the session/dose arm of the study unless this is required for safety reasons;
2) The committee's judgments as to whether participants' safety, privacy, and confidentiality has been consistently assured by the investigators;
3) Whether the DSMB recommends an interim analysis pertinent to evaluating participants' safety;
4) Judgments as to whether research instruments have been administered in a uniform manner and in a way that maintains participants' confidentiality;
5) The committee's review of the study's progress toward recruitment goals, quality of data (e.g., appropriate completion of forms), medication and psychosocial treatment adherence, and participant retention/attrition rates;
6) Its review of new scientific literature pertinent to the safety of participants or the ethics of research participation (for example, new therapeutic developments).

There will be regular, ongoing communication between the PI, the IRB, and the DSMB. IRB protocols and informed consent documents will be annually reviewed by the IRB; they will also be annually reviewed by the DSMB. The PI will take responsibility for reporting any serious adverse event (SAE) to their local IRB within 24 hours according to standard regulations. A SAE is defined as follows: death, life-threatening adverse event, inpatient hospitalization, persistent or significant disability/incapacity, and medically significant event. The PI will also be responsible for reporting any SAEs to the DSMB, and the NIDA Project Officer. Actions taken by the IRB in response to adverse event reports will be reported to the DSMB and the NIDA Project Officer and OHRP office.

7. Adverse event monitoring, reporting, and follow-up

7.1 Adverse event reporting and harms

An Adverse Event (AE) is defined as any medical occurrence in a participant, including any abnormal sign (e.g., abnormal physical exam or laboratory finding), symptom, or disease, temporally associated with the participant's involvement in the research, whether or not considered related to participation in the research. This definition includes concurrent illnesses or injuries and exacerbation of pre-existing conditions. The research team will collect AEs during study visits from Enrollment through Study

Termination. Participants will be asked directly how they are feeling during each contact, and AEs may be captured spontaneously during drug administration sessions, telephone calls, or other correspondence. Completed measures may create suspicion that an AE occurred; in this case, the site staff should follow-up with the participant. All AEs will be monitored by the study team until resolution or, if the AE becomes chronic, a cause can be identified.

Adverse events will be monitored for each participant at the time of enrollment and reported to the Stanford IRB at the continuing review, yearly. Unexpected deaths or life-threatening experiences related to the research must be reported to the Stanford IRB within 5 working days from the PD learning of the event.

Events and information which require prompt reporting the Stanford IRB

1. Unanticipated problems (UPs) involving risks to participants or others

   Events (internal or external, deaths, life-threatening experiences, injuries, or other) occurring during the research study, which in the opinion of the Monitoring Entity or the PD meet all of the following criteria:

   - Unexpected

     in terms of nature, severity, or frequency, given (a) the research procedures described in the protocol-related documents, and (b) the characteristics of the subject population being studied;

   AND

   - Related to participation in the research or there is a reasonable possibility or likelihood

     that the incident, experience, or outcome may have been caused by the procedures involved in the research;

   AND

   - Harmful

     the research places subjects or others at a greater risk of harm (including physical, psychological, economic, or social harm) than was previously known or recognized.

2. New Information that indicates a change to the risks or potential benefits of the research in terms of severity or frequency or impacts the subject's willingness to participate (e.g., DSMB/DSMC Report, other safety information or publication, suspension or premature termination by the sponsor or investigator).
3. Noncompliance: An action or activity in human subject research at variance with the approved IRB protocol, other requirements and determinations of the IRB, the HRPP Policy Manual and other applicable policies of Stanford University, SHC, LPCH, VAPAHCS (e.g., VHA Handbook 1200.5), Palo Alto Veterans Institute for Research (PAVIR) or relevant state or federal laws.
   a. When the event is:

      i. Possibly serious

- Noncompliance that affects the rights or welfare of human subject research participants.

ii. Possibly continuous

- A pattern of noncompliance that continues to occur after a report of noncompliance and a corrective action plan has been reviewed and approved by the IRB, after an investigator has been warned to correct errors or noncompliance, or a circumstance in which an investigator fails to cooperate with investigating or correcting non-compliance.

4. Complaint unresolved by the research team.
5. Incarceration when in the opinion of the PD it is in the best interest of the participant to remain in the study.

If an AE is unresolved when a participant terminates from the study, a clinical assessment will be made by the site clinician and/or investigator as to whether continued follow-up of the AE is warranted. The severity of events reported on the "Adverse Events" will be determined by the site clinician as:

- Mild: No limitation in normal daily activity
- Moderate: Some limitation in normal daily activity
- Severe: Unable to perform normal daily activity

The relationship of each AE to the drug administration will be determined via analysis and the opinion of the investigator will be recorded at the time the event was recorded. Adverse events will be recorded for the duration of a participant's active enrollment in the study and reported to the Stanford IRB upon continuing review, once a year.

7.2 Follow-up report

If an SAE has not resolved at the time of the initial report and new information arises that changes the investigator's assessment of the event, a follow-up report including all relevant new or reassessed information (e.g., concomitant medication, medical history) should be submitted to the IRB. The investigator is responsible for ensuring that all SAE are followed until either resolved or stable.

8. Internal audit, protocol amendment, and study dissemination plans

8.1 Frequency and plans for auditing trial conduct

We have established an internal auditing process to ensure study compliance is followed. A nominated study monitor will conduct a bi-annual audit outlined in the clinical monitoring plan for this study. The study monitor will collect study essential files including but not limited to; IRB-approved study material, study visit documentation, study drug logs, NIH annual progress report, and DSMB Bi-Annual reports. Once reviewed, the study monitor will work with the study coordinator to ensure all items to be corrected are addressed and corrected. This monitoring plan will occur at study initiation, bi-annually during study execution, and at the end of the study.

8.2 Plans for communicating important protocol amendments to relevant parties (e.g., trial participants, ethical committees)

Upon important protocol amendments this information will be communicated via email effectively to all necessary parties. These parties include but are not limited to the DSMB, the IRB, study staff, study participants, sponsors, and anyone else that necessitates this kind of information. If follow-up is required, study staff and the PI will employ a meeting to ensure clarity on the protocol amendment.

8.3 Dissemination plans

The plan to disseminate results will be via publications in scientific journals available to the public. All participants will be on mailing lists and informed of when a paper is published.

References

1. Hamilton M. The assessment of anxiety states by rating. *British journal of medical psychology*. 1959;
2. Hamilton M. A rating scale for depression. *Journal of neurology, neurosurgery, and psychiatry*. 1960;23(1):56.
3. Sheehan DV, Lecrubier Y, Sheehan KH, et al. The Mini-International Neuropsychiatric Interview (MINI): the development and validation of a structured diagnostic psychiatric interview for DSM-IV and ICD-10. *Journal of clinical psychiatry*. 1998;59(20):22-33.
4. Knutson B, Westdorp A, Kaiser E, Hommer D. FMRI visualization of brain activity during a monetary incentive delay task. *Neuroimage*. 2000;12(1):20-27.
5. Korgaonkar MS, Grieve SM, Etkin A, Koslow SH, Williams LM. Using standardized fMRI protocols to identify patterns of prefrontal circuit dysregulation that are common and specific to cognitive and emotional tasks in major depressive disorder: first wave results from the iSPOT-D study. *Neuropsychopharmacology*. 2013;38(5):863-871.
6. Saunders JB, Aasland OG, Babor TF, De la Fuente JR, Grant M. Development of the alcohol use disorders identification test (AUDIT): WHO collaborative project on early detection of persons with harmful alcohol consumption-II. *Addiction*. 1993;88(6):791-804.
7. Wang Y-P, Gorenstein C. Psychometric properties of the Beck Depression Inventory-II: a comprehensive review. *Brazilian Journal of Psychiatry*. 2013;35:416-431.
8. Beck AT, Epstein N, Brown G, Steer RA. An inventory for measuring clinical anxiety: psychometric properties. *Journal of consulting and clinical psychology*. 1988;56(6):893.
9. Carver CS, White TL. Behavioral inhibition, behavioral activation, and affective responses to impending reward and punishment: the BIS/BAS scales. *Journal of personality and social psychology*. 1994;67(2):319.
10. Adamson SJ, Kay-Lambkin FJ, Baker AL, et al. An improved brief measure of cannabis misuse: the Cannabis Use Disorders Identification Test-Revised (CUDIT-R). *Drug and alcohol dependence*. 2010;110(1-2):137-143.
11. Patton JH, Stanford MS, Barratt ES. Factor structure of the Barratt impulsiveness scale. *Journal of clinical psychology*. 1995;51(6):768-774.
12. Oldfield RC. The assessment and analysis of handedness: the Edinburgh inventory. *Neuropsychologia*. 1971;9(1):97-113.
13. Diener E, Emmons RA, Larsen RJ, Griffin S. The satisfaction with life scale. *Journal of personality assessment*. 1985;49(1):71-75.
14. Carver CS. You want to measure coping but your protocol'too long: Consider the brief cope. *International journal of behavioral medicine*. 1997;4(1):92-100.
15. Lovibond S. Manual for the depression anxiety stress scales. *Psychology Foundation of Australia*. 1995;
16. McFARLANE A, Clark CR, Bryant RA, et al. The impact of early life stress on psychophysiological, personality and behavioral measures in 740 non-clinical subjects. *Journal of integrative neuroscience*. 2005;4(01):27-40.
17. Gross JJ, John OP. Individual differences in two emotion regulation processes: implications for affect, relationships, and well-being. *Journal of personality and social psychology*. 2003;85(2):348.
18. Spitzer RL, Kroenke K, Williams JB, Löwe B. A brief measure for assessing generalized anxiety disorder: the GAD-7. *Archives of internal medicine*. 2006;166(10):1092-1097.
19. Kessler RC, Barber C, Beck A, et al. The world health organization health and work performance questionnaire (HPQ). *Journal of occupational and environmental medicine*. 2003;45(2):156-174.

20. Organization WH. *WHOQOL-BREF: introduction, administration, scoring and generic version of the assessment: field trial version, December 1996*. 1996.
21. Fagerstrom KO, Heatherton TF, Kozlowski LT. Nicotine addiction and its assessment. *Ear, nose, & throat journal*. 1990;69(11):763-765.
22. Watson D, Weber K, Assenheimer JS, Clark LA, Strauss ME, McCormick RA. Testing a tripartite model: I. Evaluating the convergent and discriminant validity of anxiety and depression symptom scales. *Journal of abnormal psychology*. 1995;104(1):3.
23. Watson D, Clark LA, Tellegen A. Development and validation of brief measures of positive and negative affect: the PANAS scales. *Journal of personality and social psychology*. 1988;54(6):1063.
24. Weathers F, Huska J, Keane T. The PTSD Checklist-Civilian Version (PCL-C). Available from FW Weathers. *National Center for PTSD, Boston Veterans Affairs Medical Center*. 1991;150:02130.
25. Kroenke K, Spitzer RL, Williams JB. The PHQ-9: validity of a brief depression severity measure. *Journal of general internal medicine*. 2001;16(9):606-613.
26. Yao B, Sripada RK, Klumpp H, et al. Penn State Worry Questionnaire–10: A new tool for measurement-based care. *Psychiatry Research*. 2016;239:62-67.
27. Rush AJ, Trivedi MH, Ibrahim HM, et al. The 16-Item Quick Inventory of Depressive Symptomatology (QIDS), clinician rating (QIDS-C), and self-report (QIDS-SR): a psychometric evaluation in patients with chronic major depression. *Biological psychiatry*. 2003;54(5):573-583.
28. Group WAW. The alcohol, smoking and substance involvement screening test (ASSIST): development, reliability and feasibility. *Addiction*. 2002;97(9):1183-1194.
29. Treynor W, Gonzalez R, Nolen-Hoeksema S. Rumination reconsidered: A psychometric analysis. *Cognitive therapy and research*. 2003;27:247-259.
30. Hennessy CH, Moriarty DG, Zack MM, Scherr PA, Brackbill R. Measuring health-related quality of life for public health surveillance. *Public health reports*. 1994;109(5):665.
31. Blevins CA, Weathers FW, Davis MT, Witte TK, Domino JL. The posttraumatic stress disorder checklist for DSM-5 (PCL-5): Development and initial psychometric evaluation. *Journal of traumatic stress*. 2015;28(6):489-498.
32. Davis MH. Measuring individual differences in empathy: Evidence for a multidimensional approach. *Journal of personality and social psychology*. 1983;44(1):113.
33. John OP, Donahue EM, Kentle RL. Big five inventory. *Journal of personality and social psychology*. 1991;
34. Juckel G. Personal and social performance scale. *Encyclopedia of Quality of Life and Well-Being Research*. 2014:4719-4724.
35. Bremner JD, Krystal JH, Putnam FW, et al. Measurement of dissociative states with the clinician-administered dissociative states scale (CADSS). *Journal of traumatic stress*. 1998;11:125-136.
36. Partridge C, Johnston M. Perceived control of recovery from physical disability: Measurement and prediction. *British journal of clinical psychology*. 1989;28(1):53-59.
37. Dittrich A, Lamparter D, Maurer M. *5D-ASC: Questionnaire for the assessment of altered states of consciousness. A Short Introduction.* 3rd ed. PSIN PLUS; 2010.
38. Dittrich A, Lamparter D, Maurer M. *5D-ABZ: Fragebogen zur Erfassung Aussergewöhnlicher Bewusstseinszustände. Eine kurze Einführung [5D-ASC: Questionnaire for the assessment of altered states of consciousness. A short introduction]*. PSIN PLUS; 2006.
39. Hayes M. Experimental development of the graphic rating method. *Psychological Bulletin*. 1921;18:98-99.
40. Lovibond PF, Lovibond SH. The structure of negative emotional states: Comparison of the Depression Anxiety Stress Scales (DASS) with the Beck Depression and Anxiety Inventories. *Behaviour research and therapy*. 1995;33(3):335-343.

41. Williams LM, Cooper NJ, Wisniewski SR, et al. Sensitivity, specificity, and predictive power of the "Brief Risk-resilience Index for SCreening," a brief pan-diagnostic web screen for emotional health. *Brain Behav*. Sep 2012;2(5):576-89. doi:10.1002/brb3.76
42. Snaith RP, Hamilton M, Morley S, Humayan A, Hargreaves D, Trigwell P. A scale for the assessment of hedonic tone the Snaith-Hamilton Pleasure Scale. *Br J Psychiatry*. Jul 1995;167(1):99-103. doi:10.1192/bjp.167.1.99
43. Radakovic R, Abrahams S. Developing a new apathy measurement scale: Dimensional Apathy Scale. *Psychiatry Res*. Nov 30 2014;219(3):658-63. doi:10.1016/j.psychres.2014.06.010
44. Llerena K, Park SG, McCarthy JM, Couture SM, Bennett ME, Blanchard JJ. The Motivation and Pleasure Scale-Self-Report (MAP-SR): reliability and validity of a self-report measure of negative symptoms. *Compr Psychiatry*. Jul 2013;54(5):568-74. doi:10.1016/j.comppsych.2012.12.001
45. Rizvi SJ, Quilty LC, Sproule BA, Cyriac A, Bagby RM, Kennedy SH. Development and validation of the Dimensional Anhedonia Rating Scale (DARS) in a community sample and individuals with major depression. *Psychiatry research*. 2015;229(1-2):109-119.
46. Leucht S, Kane JM, Kissling W, Hamann J, Etschel E, Engel R. Clinical implications of Brief Psychiatric Rating Scale scores. *Br J Psychiatry*. Oct 2005;187:366-71. doi:10.1192/bjp.187.4.366
47. Foulkes L, Viding E, McCrory E, Neumann CS. Social Reward Questionnaire (SRQ): development and validation. *Frontiers in psychology*. 2014;5:201.
48. Wolff M, Mertens LJ, Walter M, Enge S, Evens R. The Acceptance/Avoidance-Promoting Experiences Questionnaire (APEQ): A theory-based approach to psychedelic drugs' effects on psychological flexibility. *Journal of Psychopharmacology*. 2022;36(3):387-408.
49. Mathersul D, Palmer DM, Gur RC, et al. Explicit identification and implicit recognition of facial emotions: II. Core domains and relationships with general cognition. *J Clin Exp Neuropsychol*. Apr 2009;31(3):278-91. doi:10.1080/13803390802043619
50. Rowe DL, Cooper NJ, Liddell BJ, Clark CR, Gordon E, Williams LM. Brain structure and function correlates of general and social cognition. *J Integr Neurosci*. Mar 2007;6(1):35-74. doi:10.1142/s021963520700143x
51. Silverstein SM, Berten S, Olson P, et al. Development and validation of a World-Wide-Web-based neurocognitive assessment battery: WebNeuro. *Behav Res Methods*. Nov 2007;39(4):940-9. doi:10.3758/bf03192989
52. Goldstein-Piekarski AN, Ball TM, Samara Z, et al. Mapping Neural Circuit Biotypes to Symptoms and Behavioral Dimensions of Depression and Anxiety. *Biol Psychiatry*. Mar 15 2022;91(6):561-571. doi:10.1016/j.biopsych.2021.06.024
53. Tozzi L, Zhang X, Pines A, et al. Personalized brain circuit scores identify clinically distinct biotypes in depression and anxiety. *Nature Medicine*. 2024/07/01 2024;30(7):2076-2087. doi:10.1038/s41591-024-03057-9
54. Goldstein-Piekarski AN, Wielgosz J, Xiao L, et al. Early changes in neural circuit function engaged by negative emotion and modified by behavioural intervention are associated with depression and problem-solving outcomes: A report from the ENGAGE randomized controlled trial. *Ebiomedicine*. May 2021;67:103387. doi:10.1016/j.ebiom.2021.103387
55. Yarkoni T, Poldrack RA, Nichols TE, Van Essen DC, Wager TD. Large-scale automated synthesis of human functional neuroimaging data. *Nat Methods*. Jun 26 2011;8(8):665-70. doi:10.1038/nmeth.1635
56. Tzourio-Mazoyer N, Landeau B, Papathanassiou D, et al. Automated anatomical labeling of activations in SPM using a macroscopic anatomical parcellation of the MNI MRI single-subject brain. *Neuroimage*. Jan 2002;15(1):273-89. doi:10.1006/nimg.2001.0978
57. Tziortzi AC, Searle GE, Tzimopoulou S, et al. Imaging dopamine receptors in humans with [11C]-(+)-PHNO: dissection of D3 signal and anatomy. *Neuroimage*. Jan 1 2011;54(1):264-77. doi:10.1016/j.neuroimage.2010.06.044

58. Korgaonkar MS, Ram K, Williams LM, Gatt JM, Grieve SM. Establishing the resting state default mode network derived from functional magnetic resonance imaging tasks as an endophenotype: A twins study. *Hum Brain Mapp*. Aug 2014;35(8):3893-902. doi:10.1002/hbm.22446
59. Fan L, Li H, Zhuo J, et al. The Human Brainnetome Atlas: A New Brain Atlas Based on Connectional Architecture. *Cereb Cortex*. Aug 2016;26(8):3508-26. doi:10.1093/cercor/bhw157
60. Gordon EM, Laumann TO, Adeyemo B, Huckins JF, Kelley WM, Petersen SE. Generation and Evaluation of a Cortical Area Parcellation from Resting-State Correlations. *Cereb Cortex*. Jan 2016;26(1):288-303. doi:10.1093/cercor/bhu239
61. Fornito A, Harrison BJ, Zalesky A, Simons JS. Competitive and cooperative dynamics of large-scale brain functional networks supporting recollection. *Proc Natl Acad Sci U S A*. Jul 31 2012;109(31):12788-93. doi:10.1073/pnas.1204185109
62. He Y, Evans A. Graph theoretical modeling of brain connectivity. *Curr Opin Neurol*. Aug 2010;23(4):341-50. doi:10.1097/WCO.0b013e32833aa567
63. Alonso Martinez S, Deco G, Ter Horst GJ, Cabral J. The Dynamics of Functional Brain Networks Associated With Depressive Symptoms in a Nonclinical Sample. *Front Neural Circuits*. 2020;14:570583. doi:10.3389/fncir.2020.570583
64. Mitchell JM, Bogenschutz M, Lilienstein A, et al. MDMA-assisted therapy for severe PTSD: a randomized, double-blind, placebo-controlled phase 3 study. *Nat Med*. Jun 2021;27(6):1025-1033. doi:10.1038/s41591-021-01336-3
65. Mitchell JM, Ot'alora GM, van der Kolk B, et al. MDMA-assisted therapy for moderate to severe PTSD: a randomized, placebo-controlled phase 3 trial. *Nat Med*. Oct 2023;29(10):2473-2480. doi:10.1038/s41591-023-02565-4
66. Bedi G, Phan KL, Angstadt M, de Wit H. Effects of MDMA on sociability and neural response to social threat and social reward. *Psychopharmacology (Berl)*. Nov 2009;207(1):73-83. doi:10.1007/s00213-009-1635-z
67. Papaseit E, Torrens M, Perez-Mana C, Muga R, Farre M. Key interindividual determinants in MDMA pharmacodynamics. *Expert Opin Drug Metab Toxicol*. Feb 2018;14(2):183-195. doi:10.1080/17425255.2018.1424832
68. Liechti ME, Gamma A, Vollenweider FX. Gender differences in the subjective effects of MDMA. *Psychopharmacology (Berl)*. Mar 1 2001;154(2):161-8. doi:10.1007/s002130000648
69. Gamma A, Buck A, Berthold T, Liechti ME, Vollenweider FX. 3,4-Methylenedioxymethamphetamine (MDMA) modulates cortical and limbic brain activity as measured by [H(2)(15)O]-PET in healthy humans. *Neuropsychopharmacol*. Oct 2000;23(4):388-95. doi:10.1016/s0893-133x(00)00130-5
