# Supplementary material for: Negative Affect Circuit Subtypes and Neural, Behavioral, and Affective Responses to MDMA: A Randomized Clinical Trial
Source: JAMA Netw Open. 2025 Apr 30;8(4):e257803. doi: 10.1001/jamanetworkopen.2025.7803 (PMC12044494; doi:10.1001/jamanetworkopen.2025.7803)
Supplement: Supplement 2. — eMethods. Prior Power Analysis, Participants, and Assessments eResults. Multiple Imputation Analysis for Imaging Data eTable 1. Summary of the Linear Mixed Model for Right Amygdala Activity eTable 2. Summary of the Linear Mixed Model for Left Amygdala Activity eTable 3. Summary of the Linear Mixed Model for sgACC Activity eTable 4. Summary of the Linear Mixed Model for sgACC to Right Amygdala Connectivity eTable 5. Summary of the Linear Mixed Model for Implicit Threat Bias eTable 6. Summary of the Linear Mixed Model for Threat Face Likability eTable 7. Summary of the Linear Mixed Model for VAS Wanting to Be With Other People eTable 8. Summary of the Linear Mixed Model for VAS Feeling Secure eTable 9. Summary of the Linear Mixed Model for Anxiety eTable 10. Summary of the Linear Mixed Model for Impaired Control and Cognition eTable 11. Summary of the Linear Mixed Model for Right Amygdala Activity With Multiple Imputation eTable 12. Summary of the Linear Mixed Model for sgACC Activity With Multiple Imputation eTable 13. Summary of the Linear Mixed Model for sgACC to Right Amygdala Connectivity With Multiple Imputation eTable 14. Summary of Participants’ Neural, Behavioral, and Affective Changes and Quotations After Administration of 120 mg of MDMA vs Placebo eTable 15. Accuracy of Dose Identification Across Different Drug Conditions eFigure 1. Study Design and Baseline Stratification for the Randomized Controlled Mechanistic MDMA Trial eFigure 2. Baseline Clinical Symptoms for High (NTNA+) and Low (NTNA−) Negative Affect Circuit Activity Subgroups Evoked by Nonconscious Threat eFigure 3. Acute Neural, Behavioral, and Affective Response to Placebo and MDMA at 80 mg and 120 mg for High (NTNA+) and Low (NTNA−) Negative Affect Circuit Activity Subgroups Evoked by Nonconscious Threat eReferences. [file jamanetwopen-e257803-s002.pdf]

Supplementary Online Content

Zhang X, Hack LM, Bertrand C, et al. Negative affect circuit subtypes and neural, behavioral, and affective responses to MDMA: a randomized clinical trial. *JAMA Netw Open.* 2025;8(4):e257803. doi:10.1001/jamanetworkopen.2025.7803

eMethods. Prior Power Analysis, Participants, and Assessments
eResults. Multiple Imputation Analysis for Imaging Data
eTable 1. Summary of the Linear Mixed Model for Right Amygdala Activity
eTable 2. Summary of the Linear Mixed Model for Left Amygdala Activity
eTable 3. Summary of the Linear Mixed Model for sgACC Activity
eTable 4. Summary of the Linear Mixed Model for sgACC to Right Amygdala Connectivity
eTable 5. Summary of the Linear Mixed Model for Implicit Threat Bias
eTable 6. Summary of the Linear Mixed Model for Threat Face Likability
eTable 7. Summary of the Linear Mixed Model for VAS Wanting to Be With Other People
eTable 8. Summary of the Linear Mixed Model for VAS Feeling Secure
eTable 9. Summary of the Linear Mixed Model for Anxiety
eTable 10. Summary of the Linear Mixed Model for Impaired Control and Cognition
eTable 11. Summary of the Linear Mixed Model for Right Amygdala Activity With Multiple Imputation
eTable 12. Summary of the Linear Mixed Model for sgACC Activity With Multiple Imputation
eTable 13. Summary of the Linear Mixed Model for sgACC to Right Amygdala Connectivity With Multiple Imputation
eTable 14. Summary of Participants’ Neural, Behavioral, and Affective Changes and Quotations After Administration of 120 mg of MDMA vs Placebo
eTable 15. Accuracy of Dose Identification Across Different Drug Conditions
eFigure 1. Study Design and Baseline Stratification for the Randomized Controlled Mechanistic MDMA Trial
eFigure 2. Baseline Clinical Symptoms for High (NTN_{A+}) and Low (NTN_{A-}) Negative Affect Circuit Activity Subgroups Evoked by Nonconscious Threat
eFigure 3. Acute Neural, Behavioral, and Affective Response to Placebo and MDMA at 80 mg and 120 mg for High (NTN_{A+}) and Low (NTN_{A-}) Negative Affect Circuit Activity Subgroups Evoked by Nonconscious Threat
eReferences.

This supplementary material has been provided by the authors to give readers additional information about their work.

© 2025 Zhang X et al. *JAMA Network Open*.

eMethods. Prior Power Analysis, Participants, and Assessments

Prior power analysis
To estimate the sample size for detecting the effect of 3,4-methylenedioxymethamphetamine (MDMA) on our primary neural measures of the negative affect circuit, we drew on prior reported effect sizes from a study in which imaging was similarly undertaken immediately following administration of MDMA in a repeated measures design[1]. In this prior study, the effect size reported for amygdala activity was $\eta_p^2 = 0.546$, which could be converted to the Cohen f of 0.67 for power analysis. We conducted a power analysis using G*Power Version 3.1.9.663 for sample size estimation. The sample size needed to detect an effect on neural activity with $\alpha = .05$ and at least 95% power for a within-subjects design with three repeated imaging measurements is N = 8. Therefore, the obtained sample size of N = 16 is adequate to detect MDMA's dose-dependent effects on primary measures of interest. However, no previous studies have reported subgroup differences in MDMA-induced effects, and therefore no formal sample size estimation was performed for between-subgroup comparisons in this study.

Participants
The study received approval from the Stanford Institutional Review Board (IRB #52244), the FDA (Investigational New Drug #153746), and the Research Advisory Panel of California. Additionally, the U.S. Drug Enforcement Administration approved the study after an inspection of the installed safe and drug handling procedures, with L.M.H. designated as the Schedule I license holder. The first participant was enrolled on November 15, 2021 and the last participant was enrolled on September 30, 2022. Data were collected using REDCap versions 11.4.4 through 12.5.11. Participants were recruited through Facebook Ads using Institutional Review Board-approved material. Individuals who expressed interest in the study were directed to an online screening survey in REDCap. Individuals who were eligible to participate were contacted by a research coordinator for a telephone screening. On this telephone call, the research coordinator provided the individual with additional information about the study, obtained informed consent**,** collected additional demographic information, and scheduled an in-person screening visit at a research clinic. Screening procedures were conducted by trained medical professionals, including phlebotomists (blood draw), research nurses (electrocardiogram and vital signs), trained research coordinators (drug and psychiatric histories; urine drug and pregnancy tests), and licensed study clinicians (physical examinations and confirmation of psychiatric histories). Baseline and drug session procedures were conducted by trained research coordinators (study assessments and scanning) and licensed study clinicians (drug administration, monitoring, and safety assessments). Travel was arranged for participants to all drug visits, and they were financially compensated for their participation in the study.

Visits
Given the within-participants design of the study, each participant received all three of the specified doses across the duration of the trial. After the baseline visit, each drug visit was separated from any other drug visit by 10-14 days to avoid drug carry-over effects. In each drug visit, participants arrived fasted in the morning to minimize the risk of emesis. They completed a urine drug screen and pregnancy test (if applicable) and had their baseline vitals recorded at the Stanford Center for Cognitive and Neurobiological Imaging, where they were met by the study clinician. Participants were instructed to orally ingest three pills with consumption of an electrolyte solution. For safety reasons, pulse, blood pressure, temperature, and oxygen saturation were monitored and recorded every 30 minutes for all conditions throughout the drug visit, including the scanning sessions. The total visit time for drug visits was 6-8 hours.

Assessments of the negative affect circuit using nonconscious Facial Expressions of Emotion Task (FEET) fMRI
Structural Magnetic Resonance Imaging (MRI)
Imaging data were acquired on a GE Discovery UHP 3T scanner using a Nova Medical 32-channel head coil at the Stanford Center for Cognitive and Neurobiological Imaging. A T1-weighted and a T2-weighted anatomical MRI image was collected at the baseline visit for normalization of all functional MRI (fMRI) data into standard space. The T1-weighted MRI was collected in sagittal orientation with TR = 3.0 s, TE = 3.548ms, FA = 8º, acquisition time = 8:33, field of view (FOV) = 256 × 256mm, 3D matrix size = 320 × 320 × 230, voxel size = 0.8 mm isotropic, motion correction = PROMO. The T2-weighted MRI was collected in sagittal orientation with TR = 2.5 s, TE = Maximum, FA = 90, acquisition time = 5:42, FOV = 256 × 256mm, 3D matrix size = 320 × 320 × 216, voxel size = 0.8mm isotropic, motion correction = PROMO.

© 2025 Zhang X et al. *JAMA Network Open*.

Functional Magnetic Resonance Imaging (fMRI)
BOLD fMRI was acquired with axial slices in an interleaved order, with TR = 2s, TE = 27.5ms, FA = 77°, acquisition time = 5:08, FOV = 222 x 222mm, voxel size = 3mm isotropic. During the scan, participants nonconsciously viewed facial expressions of emotion, during which fMRI data were collected, to probe automatic bottom-up activity of the negative and positive affect circuits[2]. Stimuli were from a standardized series of facial expressions, including threat-related emotions (fear, anger), reward-related emotions (happy), and loss-related emotions (sad), along with neutral expressions. Stimuli were modified such that the eyes were presented in the central position of the image. Each face was presented for 16.7ms (1 monitor frame), followed immediately by a neutral face perceptual mask for 150.3ms (9 monitor frames) and an interstimulus interval of 1083ms. Neutral masked faces stimuli were offset slightly by 1 degree in random directions to control for the possible detection of emotions based on perceptual features (e.g., the apparent motion in the pairing of a fear face with upraised eyebrows followed by a neutral face mask compared with the pairing of an angry face with contracted eyebrows followed by a neutral face mask). Using behavioral psychophysiological testing, we have shown that when faces in this paradigm are presented at ≤ 20ms, they meet signal detection criteria for being at the subliminal threshold for detection such that individual participants cannot consciously detect the presence of the face nor discriminate the facial expression[3]. A total of 240 masked emotional faces from six different emotions were presented, with eight faces of the same emotion forming a block and each emotion block repeated for five times in a pseudorandom order. Participants were instructed to press a button when seeing a face, and we controlled for active attention by monitoring alertness with an eye tracking system. One hundred fifty-four volumes were acquired, with the first three volumes cut to account for the non-steady state.

Image preprocessing and quality control procedure
All participants' data were quantified using the Stanford Et Cere Image Processing System protocol based on the version two of PanLab Imaging Pipeline (PLIP)[4]. An fMRI preprocessing pipeline was completed using SPM8 (https://www.fil.ion.ucl.ac.uk/spm/software/spm8/) and FSL 5.0.9 (https://fsl.fmrib.ox.ac.uk/fsl) following previously established procedures[4,5], including realignment, co-registration, normalization to the standard Montréal Neurological Institute space, and smoothing with a Gaussian kernel of 8mm full width at half maximum. A participant's data were included if no more than 25% of time points (38 out of 151 frames) were censored by frame-wise displacement ≥ 0.3mm or scaled signal intensity differences ≥ 10. A temporal mask was then created for each censored volume and its subsequent volume and was used as nuisance regressors in the participant-level modeling. Additional quality control diagnostics included visual inspection of the raw fMRI timeseries for artifacts and signal dropout. One participant dropped out at the baseline visit before any drug visits because of claustrophobia-induced panic attack. This resulted in a total of N = 16, 13, and 9, for the Facial Expressions of Emotion Task (FEET) imaging data at baseline, 80 mg of MDMA, and 120 mg of MDMA, respectively.

FEET task analysis
To compute task-evoked activations, the onset times of emotion stimuli were convolved with a canonical hemodynamic response function and entered into a general linear model analysis as regressors of interest, and the six realignment parameters were entered as regressors of no interest. A high-pass filter with a cutoff period of 128 seconds was applied. Activation maps for threat (fear and anger facial expressions) relative to neutral faces were estimated. For functional connectivity estimation, a psychophysiological interaction (PPI) approach was used for the contrast of threat versus neutral. Specifically, the psychological variable (task contrast of interest; threat versus neutral), the physiological variable (deconvolved time course of the first eigenvariate of that region of interest's (ROI's) time course), and the interaction between the psychological and physiological variables (PPI effect of interest) were entered into the general linear model, as were motion parameters. Functional connectivity PPI maps for threat relative to neutral faces were estimated for the seed region of the subgenual anterior cingulate cortex (sgACC).

Definition of region of interest
The *a priori* ROIs—the bilateral amygdala and sgACC—were defined and validated as described previously using the same nonconscious threat stimuli[4]. Briefly, a search was conducted for 'threat' in the meta-analytic database Neurosynth[6]. The resulting uniformity map was thresholded at false discovery rate < 0.01, peak coordinates of the resulting clusters were identified, and voxels at a maximum of 10mm from these peaks were used to generate regions of interest. For the sgACC, the peak Montreal Neurological Institute coordinates were 4, 26, -10. For the bilateral amygdala, the Neurosynth map was intersected with anatomically defined boundaries from the Automated Anatomical Labelling atlas[7]. These ROI masks were then intersected with each participant's gray matter mask to

© 2025 Zhang X et al. *JAMA Network Open*.

ensure specificity to the gray matter anatomy of each participant. ROI masks were also ensured to pass quality controls for sufficient gray matter overlap, temporal signal-to-noise ratio, and internal consistency of functional connectivity.

<u>Derivation of negative affect circuit scores, referenced to a healthy norm</u>

Activity and connectivity of the amygdala and sgACC that defined the negative affect circuit were extracted for the contrast of threat versus neutral and were expressed in standard deviation units relative to the mean and standard deviation of a sample of healthy controls that underwent the same task (z-scores). The only variation in the task setup is that, in our current study, participants were instructed to press a button upon seeing a face to maintain attention levels. This standardization approach allows us to interpret the direction and magnitude of the measured neural activity as standard deviation unit distance from a healthy reference mean, derived from a reference dataset without subclinical symptoms, trauma exposure, or prior MDMA use. This resulted in the following negative affect circuit measures for further analyses: the activity of bilateral amygdala and sgACC and the connectivity between sgACC and bilateral amygdala if their activity is modulated by MDMA. We previously demonstrated that this system produces valid and clinically useful individual circuit clinical scores[4,8].

Assessments of behavioral responses

We used an implicit recognition of facial emotion test in the WebNeuro battery to assess nonconscious processing of facial emotion in the context of memory recognition. Participants made the "NEW versus OLD" face recognition decision of whether they had seen the face approximately 20 minutes earlier or not, implicitly primed by emotion of expressions from the fMRI threat task, to assess the nonconscious influence of emotions. Reaction times primed by angry faces relative to neutral faces were calculated as a standardized z-score, referenced to age-matched norms from the WebNeuro battery[1,9]. Participants P013 (session 4) to P017 completed a Total Brain version of the WebNeuro battery that assessed the same task. Psychometric properties for this test have been established and include norms, construct validity, validation against traditional neuropsychological tests that evaluate equivalent functions, test-retest reliability, and consistency across cultures. Additionally, after the scan was completed, participants completed a facial likability assessment using example faces of those presented during the fMRI threat task. Participants were shown a series of faces and asked to rate their likability on a scale of 0 ("most negative reaction") to 100 ("most positive reaction").

Assessments of affective responses

To measure MDMA-induced affective responses, we utilized a custom Visual Analog Scale (VAS) and the 5-Dimensional Altered States of Consciousness (5D-ASC) rating scale[10,11] in all three drug visits immediately after the scan was completed. The VAS contains a list of words/statements that describe the feelings—with 0 meaning "not at all" and 100 meaning "extremely"—extracted from the Profile of Mood States[12] and/or from previous MDMA studies[13]. The 5D-ASC is a 94-item scale that evaluates drug-induced change in affective response or psychological functioning compared to normal waking consciousness[14]. We chose to examine "I want to be with other people" and "I feel secure" from the VAS, and "Anxiety" and "Impaired Control and Cognition" from the 5D-ASC to assess MDMA-induced positively and negatively valenced affective states, respectively. In addition to the above quantitative assessments, we used keywords from the VAS and 5D-ASC to prompt participants to describe their experience, and recorded narratives for all participants. We addressed missing data in each participant's 5D-ASC data under a certain dose condition as follows: if the missing items were fewer than 10% of the total number of items in the questionnaire, we replaced missing items with the group mean of that dose condition. This brought the sample size for 5D-ASC data to N =16 for all three drug visits.

Multiple imputation for missing imaging data

To address missing data in the 80-mg and 120-mg MDMA conditions due to excessive motions, we implemented multiple imputation using the Multivariate Imputation by Chained Equations package in R[15]. This approach enables robust estimation by accounting for uncertainty in the missing data through the creation of multiple datasets. Specifically, we generated 50 datasets with 100 iterations per imputation, using the value from baseline, placebo, as well as 80-mg and 120-mg MDMA conditions as predictors in the imputation model. This resulted in a total of N=16 for the FEET imaging data at all visits.

Multiple imputation analysis for imaging data

© 2025 Zhang X et al. *JAMA Network Open*.

After applying multiple imputation for missing data in the MDMA conditions, we re-evaluated the significant acute MDMA-induced changes in the negative affect circuit across conditions. Each imputed dataset was analyzed separately with our linear mixed model exactly the same as the non-imputed data, where negative affect circuit activity or connectivity served as the dependent variable and baseline subgroup (NTN_{A+} or NTN_{A-}), dose (placebo, 80 mg of MDMA, or 120 mg of MDMA), and subgroup-by-dose interaction as fixed effects. Finally, parameter estimates from each imputed dataset were pooled according to Rubin's Rules, providing an aggregated estimate that accounts for the variability due to imputation.

© 2025 Zhang X et al. *JAMA Network Open*.

eResults. Multiple Imputation Analysis for Imaging Data

The interaction effect between baseline subgroup and dose remained consistent in the pooled analysis, confirming that compared to the NTN_{A-} subgroup, the NTN_{A+} subgroup showed a reduction in activity in right amygdala, as well as an increase in connectivity between the sgACC and right amygdala under 120 mg of MDMA versus placebo (**eTable 11-13**).

© 2025 Zhang X et al. *JAMA Network Open*.

eTable 1. Summary of the Linear Mixed Model for Right Amygdala Activity

a. Model effects

Effect	F	Numerator df	Denominator df	p	d
Subgroup	1.10	1	14	0.31	0.56
Dose	6.32	2	18	0.01	1.68
Subgroup x Dose	4.00	2	18	0.04	1.33

Note: This table presents main effects of subgroup and dose, and subgroup-by-dose interaction, with F statistics, p values, and effect size of Cohen d converted from partial eta squared.

b. Subgroup × Dose interaction contrasts: Pairwise comparisons

Group pairwise	Dose pairwise	Estimate	SE	95% CI	df	t	p	d
NTN_{A+} – NTN_{A-}	120 mg – placebo	-1.43	0.55	[-2.60, -0.27]	18	-2.58	0.02	-1.22
NTN_{A+} – NTN_{A-}	80 mg – placebo	-0.56	0.66	[-1.95, 0.82]	18	-0.86	0.40	-0.40
NTN_{A+} – NTN_{A-}	120 mg – 80 mg	-0.87	0.51	[-1.93, 0.19]	18	-1.72	0.10	-0.81

Note: This table presents pairwise comparisons of dose effects between NTN_{A+} and NTN_{A-} subgroups, including estimated differences, SE, 95% CIs, t statistics, p values, and effect size of Cohen d.

c. Within-subgroup pairwise dose contrasts

NTN_{A+} dose contrast	Estimate	SE	95% CI	df	t	p	d
120 mg – placebo	-1.21	0.40	[-2.05, -0.37]	18	-3.04	0.01	-1.43
80 mg – placebo	0.09	0.47	[-0.90, 1.08]	18	0.19	0.85	0.09
120 mg – 80 mg	-1.30	0.37	[-2.08, -0.52]	18	-3.48	0.003	-1.64
**NTN_{A-} dose contrast**	**Estimate**	**SE**	**95% CI**	**df**	**t**	**p**	**d**
120 mg – placebo	0.23	0.39	[-0.59, 1.05]	18	0.58	0.57	0.27
80 mg – placebo	0.65	0.46	[-0.32, 1.62]	18	1.43	0.17	0.67
120 mg – 80 mg	-0.43	0.34	[-1.14, 0.28]	18	-1.25	0.23	-0.59

Note: This table presents within-subgroup pairwise dose contrasts separately for NTN_{A+} and NTN_{A-} subgroups, including estimated differences, SE, 95% CIs, t values, p values, and effect size of Cohen d.

d. Within-subgroup dose estimates

NTN_{A+} subgroup				NTN_{A-} subgroup			
**Dose**	**Estimate**	**SE**	**95% CI**	**Dose**	**Estimate**	**SE**	**95% CI**
Placebo	0.31	0.35	[-0.44, 1.06]	Placebo	-0.11	0.35	[-0.86, 0.64]
80 mg	0.40	0.32	[-0.29, 1.09]	80 mg	0.54	0.30	[-0.09, 1.18]
120 mg	-0.9	0.19	[-1.31, -0.49]	120 mg	0.12	0.17	[-0.25, 0.48]

Note: This table presents estimated means for each dose condition within the NTN_{A+} and NTN_{A-} subgroups, including estimates, SE, and 95% CIs.

Abbreviations: df, Degree of freedom adjusted using the containment method; SE, Standard error; CI, confidence interval; d, Cohen d; NTN_{A+}, High negative affect circuit activity evoked by nonconscious threat; NTN_{A-}, Low negative affect circuit activity evoked by nonconscious threat.

© 2025 Zhang X et al. *JAMA Network Open*.

eTable 2. Summary of the Linear Mixed Model for Left Amygdala Activity

a. Model effects

Effect	F	Numerator df	Denominator df	p	d
Subgroup	0.12	1	14	0.73	0.19
Dose	1.26	2	18	0.31	0.75
Subgroup x Dose	0.73	2	18	0.49	0.57

Note: This table presents main effects of subgroup and dose, and subgroup-by-dose interaction, with F statistics, p values, and effect size of Cohen d converted from partial eta squared.

b. Subgroup × Dose interaction contrasts: Pairwise comparisons

Group pairwise	Dose pairwise	Estimate	SE	95% CI	df	t	p	d
NTN_{A+} – NTN_{A-}	120 mg – placebo	-0.68	0.81	[-2.38, 1.02]	18	-0.85	0.41	-0.40
NTN_{A+} – NTN_{A-}	80 mg – placebo	0.39	0.80	[-1.3, 2.08]	18	0.48	0.64	0.23
NTN_{A+} – NTN_{A-}	120 mg – 80 mg	-1.07	0.90	[-2.96, 0.82]	18	-1.19	0.25	-0.56

Note: This table presents pairwise comparisons of dose effects between NTN_{A+} and NTN_{A-} subgroups, including estimated differences, SE, 95% CIs, t statistics, p values, and effect size of Cohen d.

c. Within-subgroup pairwise dose contrasts

NTN_{A+} dose contrast	Estimate	SE	95% CI	df	t	p	d
120 mg – placebo	-0.72	0.59	[-1.96, 0.52]	18	-1.22	0.24	-0.58
80 mg – placebo	0.53	0.58	[-0.69, 1.75]	18	0.90	0.38	0.42
120 mg – 80 mg	-1.25	0.66	[-2.64, 0.14]	18	-1.88	0.08	-0.89
**NTN_{A-} dose contrast**	**Estimate**	**SE**	**95% CI**	**df**	**t**	**p**	**d**
120 mg – placebo	-0.04	0.55	[-1.2, 1.12]	18	-0.07	0.95	-0.03
80 mg – placebo	0.14	0.55	[-1.02, 1.3]	18	0.25	0.80	0.12
120 mg – 80 mg	-0.18	0.60	[-1.44, 1.08]	18	-0.29	0.77	-0.14

Note: This table presents within-subgroup pairwise dose contrasts separately for NTN_{A+} and NTN_{A-} subgroups, including estimated differences, SE, 95% CIs, t values, p values, and effect size of Cohen d.

d. Within-subgroup dose estimates

NTN_{A+} subgroup				NTN_{A-} subgroup			
**Dose**	**Estimate**	**SE**	**95% CI**	**Dose**	**Estimate**	**SE**	**95% CI**
Placebo	0.31	0.38	[-0.51, 1.12]	Placebo	0.06	0.38	[-0.76, 0.88]
80 mg	0.83	0.50	[-0.24, 1.9]	80 mg	0.20	0.46	[-0.79, 1.19]
120 mg	-0.42	0.51	[-1.51, 0.68]	120 mg	0.02	0.46	[-0.96, 1.01]

Note: This table presents estimated means for each dose condition within the NTN_{A+} and NTN_{A-} subgroups, including estimates, SE, and 95% CIs.

Abbreviations: df, Degree of freedom adjusted using the containment method; SE, Standard error; CI, confidence interval; d, Cohen d; NTN_{A+}, High negative affect circuit activity evoked by nonconscious threat; NTN_{A-}, Low negative affect circuit activity evoked by nonconscious threat.

© 2025 Zhang X et al. *JAMA Network Open*.

eTable 3. Summary of the Linear Mixed Model for sgACC Activity

a. Model effects

Effect	F	Numerator df	Denominator df	p	d
Subgroup	0.35	1	14	0.56	0.32
Dose	2.79	2	18	0.09	1.11
Subgroup x Dose	6.21	2	18	0.01	1.66

Note: This table presents main effects of subgroup and dose, and subgroup-by-dose interaction, with F statistics, p values, and effect size of Cohen d converted from partial eta squared.

b. Subgroup × Dose interaction contrasts: Pairwise comparisons

Group pairwise	Dose pairwise	Estimate	SE	95% CI	df	t	p	d
NTN_{A+} – NTN_{A-}	120 mg – placebo	-1.48	0.45	[-2.42, -0.54]	18	-3.31	0.004	-1.56
NTN_{A+} – NTN_{A-}	80 mg – placebo	0.01	0.98	[-2.06, 2.07]	18	0.01	0.99	0.00
NTN_{A+} – NTN_{A-}	120 mg – 80 mg	-1.48	0.93	[-3.44, 0.47]	18	-1.60	0.13	-0.75

Note: This table presents pairwise comparisons of dose effects between NTN_{A+} and NTN_{A-} subgroups, including estimated differences, SE, 95% CIs, t statistics, p values, and effect size of Cohen d.

c. Within-subgroup pairwise dose contrasts

NTN_{A+} dose contrast	Estimate	SE	95% CI	df	t	p	d
120 mg – placebo	-1.07	0.33	[-1.76, -0.38]	18	-3.23	0.005	-1.52
80 mg – placebo	0.61	0.72	[-0.9, 2.12]	18	0.84	0.41	0.40
120 mg – 80 mg	-1.67	0.68	[-3.1, -0.24]	18	-2.45	0.02	-1.15
**NTN_{A-} dose contrast**	**Estimate**	**SE**	**95% CI**	**df**	**t**	**p**	**d**
120 mg – placebo	0.41	0.30	[-0.22, 1.04]	18	1.36	0.19	0.64
80 mg – placebo	0.60	0.67	[-0.81, 2.01]	18	0.90	0.38	0.42
120 mg – 80 mg	-0.19	0.63	[-1.51, 1.13]	18	-0.30	0.77	-0.14

Note: This table presents within-subgroup pairwise dose contrasts separately for NTN_{A+} and NTN_{A-} subgroups, including estimated differences, SE, 95% CIs, t values, p values, and effect size of Cohen d.

d. Within-subgroup dose estimates

NTN_{A+} subgroup				NTN_{A-} subgroup			
**Dose**	**Estimate**	**SE**	**95% CI**	**Dose**	**Estimate**	**SE**	**95% CI**
Placebo	-0.06	0.57	[-1.28, 1.16]	Placebo	-0.08	0.57	[-1.3, 1.14]
80 mg	0.54	0.85	[-1.27, 2.36]	80 mg	0.52	0.80	[-1.2, 2.24]
120 mg	-1.13	0.55	[-2.32, 0.06]	120 mg	0.33	0.54	[-0.82, 1.48]

Note: This table presents estimated means for each dose condition within the NTN_{A+} and NTN_{A-} subgroups, including estimates, SE, and 95% CIs.

Abbreviations: df, Degree of freedom adjusted using the containment method; SE, Standard error; CI, confidence interval; d, Cohen d; NTN_{A+}, High negative affect circuit activity evoked by nonconscious threat; NTN_{A-}, Low negative affect circuit activity evoked by nonconscious threat.

© 2025 Zhang X et al. *JAMA Network Open*.

eTable 4. Summary of the Linear Mixed Model for sgACC to Right Amygdala Connectivity

a. Model effects

Effect	F	Numerator df	Denominator df	p	d
Subgroup	0.15	1	14	0.71	0.21
Dose	0.59	2	18	0.56	0.51
Subgroup x Dose	3.84	2	18	0.04	1.31

Note: This table presents main effects of subgroup and dose, and subgroup-by-dose interaction, with F statistics, p values, and effect size of Cohen d converted from partial eta squared.

b. Subgroup × Dose interaction contrasts: Pairwise comparisons

Group pairwise	Dose pairwise	Estimate	SE	95% CI	df	t	p	d
NTN_{A+} – NTN_{A-}	120 mg – placebo	0.65	0.30	[0.02, 1.28]	18	2.17	0.04	1.02
NTN_{A+} – NTN_{A-}	80 mg – placebo	-0.40	0.61	[-1.68, 0.88]	18	-0.66	0.52	-0.31
NTN_{A+} – NTN_{A-}	120 mg – 80 mg	1.05	0.55	[-0.11, 2.22]	18	1.89	0.07	0.89

Note: This table presents pairwise comparisons of dose effects between NTN_{A+} and NTN_{A-} subgroups, including estimated differences, SE, 95% CIs, t statistics, p values, and effect size of Cohen d.

c. Within-subgroup pairwise dose contrasts

**NTN_{A+} dose contrast**	**Estimate**	**SE**	**95% CI**	**df**	**t**	**p**	**d**
120 mg – placebo	0.24	0.22	[-0.22, 0.7]	18	1.08	0.30	0.51
80 mg – placebo	-0.53	0.44	[-1.45, 0.39]	18	-1.18	0.25	-0.56
120 mg – 80 mg	0.76	0.41	[-0.1, 1.62]	18	1.87	0.08	0.88
**NTN_{A-} dose contrast**	**Estimate**	**SE**	**95% CI**	**df**	**t**	**p**	**d**
120 mg – placebo	-0.42	0.21	[-0.86, 0.02]	18	-2.02	0.06	-0.95
80 mg – placebo	-0.13	0.42	[-1.01, 0.75]	18	-0.31	0.76	-0.15
120 mg – 80 mg	-0.29	0.38	[-1.09, 0.51]	18	-0.77	0.45	-0.36

Note: This table presents within-subgroup pairwise dose contrasts separately for NTN_{A+} and NTN_{A-} subgroups, including estimated differences, SE, 95% CIs, t values, p values, and effect size of Cohen d.

d. Within-subgroup dose estimates

NTN_{A+} subgroup				NTN_{A-} subgroup			
**Dose**	**Estimate**	**SE**	**95% CI**	**Dose**	**Estimate**	**SE**	**95% CI**
Placebo	0.40	0.24	[-0.12, 0.91]	Placebo	0.37	0.24	[-0.15, 0.88]
80 mg	-0.13	0.43	[-1.05, 0.79]	80 mg	0.24	0.40	[-0.62, 1.1]
120 mg	0.63	0.19	[0.23, 1.03]	120 mg	-0.05	0.17	[-0.42, 0.32]

Note: This table presents estimated means for each dose condition within the NTN_{A+} and NTN_{A-} subgroups, including estimates, SE, and 95% CIs.

Abbreviations: df, Degree of freedom adjusted using the containment method; SE, Standard error; CI, confidence interval; d, Cohen d; NTN_{A+}, High negative affect circuit activity evoked by nonconscious threat; NTN_{A-}, Low negative affect circuit activity evoked by nonconscious threat.

© 2025 Zhang X et al. *JAMA Network Open*.

eTable 5. Summary of the Linear Mixed Model for Implicit Threat Bias

a. Model effects

Effect	F	Numerator df	Denominator df	p	d
Subgroup	2.96	1	14	0.11	0.92
Dose	3.18	2	27	0.06	0.97
Subgroup x Dose	1.25	2	27	0.30	0.61

Note: This table presents main effects of subgroup and dose, and subgroup-by-dose interaction, with F statistics, p values, and effect size of Cohen d converted from partial eta squared.

b. Subgroup × Dose interaction contrasts: Pairwise comparisons

Group pairwise	Dose pairwise	Estimate	SE	95% CI	df	t	p	d
NTN_{A+} – NTN_{A-}	120 mg – placebo	0.45	0.46	[-0.49, 1.39]	27	0.98	0.34	0.38
NTN_{A+} – NTN_{A-}	80 mg – placebo	0.73	0.48	[-0.26, 1.72]	27	1.51	0.14	0.58
NTN_{A+} – NTN_{A-}	120 mg – 80 mg	-0.28	0.53	[-1.38, 0.81]	27	-0.53	0.60	-0.20

Note: This table presents pairwise comparisons of dose effects between NTN_{A+} and NTN_{A-} subgroups, including estimated differences, SE, 95% CIs, t statistics, p values, and effect size of Cohen d.

c. Within-subgroup pairwise dose contrasts

NTN_{A+} dose contrast	Estimate	SE	95% CI	df	t	p	d
120 mg – placebo	0.56	0.33	[-0.12, 1.24]	27	1.69	0.10	0.65
80 mg – placebo	0.03	0.34	[-0.67, 0.73]	27	0.08	0.93	0.03
120 mg – 80 mg	0.53	0.38	[-0.25, 1.31]	27	1.39	0.18	0.54
**NTN_{A-} dose contrast**	**Estimate**	**SE**	**95% CI**	**df**	**t**	**p**	**d**
120 mg – placebo	0.11	0.32	[-0.55, 0.77]	27	0.35	0.73	0.13
80 mg – placebo	-0.70	0.34	[-1.4, 0]	27	-2.05	0.05	-0.79
120 mg – 80 mg	0.82	0.37	[0.06, 1.58]	27	2.19	0.04	0.84

Note: This table presents within-subgroup pairwise dose contrasts separately for NTN_{A+} and NTN_{A-} subgroups, including estimated differences, SE, 95% CIs, t values, p values, and effect size of Cohen d.

d. Within-subgroup dose estimates

NTN_{A+} subgroup				NTN_{A-} subgroup			
**Dose**	**Estimate**	**SE**	**95% CI**	**Dose**	**Estimate**	**SE**	**95% CI**
Placebo	-0.17	0.20	[-0.6, 0.26]	Placebo	-0.12	0.20	[-0.55, 0.31]
80 mg	-0.14	0.28	[-0.74, 0.46]	80 mg	-0.82	0.28	[-1.42, -0.23]
120 mg	0.39	0.26	[-0.18, 0.96]	120 mg	-0.01	0.25	[-0.54, 0.52]

Note: This table presents estimated means for each dose condition within the NTN_{A+} and NTN_{A-} subgroups, including estimates, SE, and 95% CIs.

Abbreviations: df, Degree of freedom adjusted using the containment method; SE, Standard error; CI, confidence interval; d, Cohen d; NTN_{A+}, High negative affect circuit activity evoked by nonconscious threat; NTN_{A-}, Low negative affect circuit activity evoked by nonconscious threat.

© 2025 Zhang X et al. *JAMA Network Open*.

eTable 6. Summary of the Linear Mixed Model for Threat Face Likability

a. Model effects

Effect	F	Numerator df	Denominator df	p	d
Subgroup	0.48	1	14	0.50	0.37
Dose	1.42	2	28	0.26	0.64
Subgroup x Dose	2.60	2	28	0.09	0.86

Note: This table presents main effects of subgroup and dose, and subgroup-by-dose interaction, with F statistics, p values, and effect size of Cohen d converted from partial eta squared.

b. Subgroup × Dose interaction contrasts: Pairwise comparisons

Group pairwise	Dose pairwise	Estimate	SE	95% CI	df	t	p	d
NTN_{A+} – NTN_{A-}	120 mg – placebo	14.38	6.31	[1.46, 27.29]	28	2.28	0.03	0.86
NTN_{A+} – NTN_{A-}	80 mg – placebo	5.38	8.43	[-11.89, 22.64]	28	0.64	0.53	0.24
NTN_{A+} – NTN_{A-}	120 mg – 80 mg	9.00	9.12	[-9.68, 27.68]	28	0.99	0.33	0.37

Note: This table presents pairwise comparisons of dose effects between NTN_{A+} and NTN_{A-} subgroups, including estimated differences, SE, 95% CIs, t statistics, p values, and effect size of Cohen d.

c. Within-subgroup pairwise dose contrasts

NTN_{A+} dose contrast	Estimate	SE	95% CI	df	t	p	d
120 mg – placebo	11.25	4.46	[2.11, 20.39]	28	2.52	0.02	0.95
80 mg – placebo	8.50	5.96	[-3.71, 20.71]	28	1.43	0.16	0.54
120 mg – 80 mg	2.75	6.45	[-10.46, 15.96]	28	0.43	0.67	0.16
**NTN_{A-} dose contrast**	**Estimate**	**SE**	**95% CI**	**df**	**t**	**p**	**d**
120 mg – placebo	-3.13	4.46	[-12.27, 6.01]	28	-0.70	0.49	-0.26
80 mg – placebo	3.13	5.96	[-9.08, 15.34]	28	0.52	0.60	0.20
120 mg – 80 mg	-6.25	6.45	[-19.46, 6.96]	28	-0.97	0.34	-0.37

Note: This table presents within-subgroup pairwise dose contrasts separately for NTN_{A+} and NTN_{A-} subgroups, including estimated differences, SE, 95% CIs, t values, p values, and effect size of Cohen d.

d. Within-subgroup dose estimates

NTN_{A+} subgroup				NTN_{A-} subgroup			
**Dose**	**Estimate**	**SE**	**95% CI**	**Dose**	**Estimate**	**SE**	**95% CI**
Placebo	12.75	6.47	[-1.12, 26.62]	Placebo	13.13	6.47	[-0.75, 27]
80 mg	21.25	7.97	[4.15, 38.35]	80 mg	16.25	7.97	[-0.85, 33.35]
120 mg	24.00	6.92	[9.15, 38.85]	120 mg	10.00	6.92	[-4.85, 24.85]

Note: This table presents estimated means for each dose condition within the NTN_{A+} and NTN_{A-} subgroups, including estimates, SE, and 95% CIs.

Abbreviations: df, Degree of freedom adjusted using the containment method; SE, Standard error; CI, confidence interval; d, Cohen d; NTN_{A+}, High negative affect circuit activity evoked by nonconscious threat; NTN_{A-}, Low negative affect circuit activity evoked by nonconscious threat.

© 2025 Zhang X et al. *JAMA Network Open*.

eTable 7. Summary of the Linear Mixed Model for VAS Wanting to Be With Other People

a. Model effects

Effect	F	Numerator df	Denominator df	p	d
Subgroup	0.05	1	14	0.83	0.12
Dose	7.98	2	28	0.00	1.51
Subgroup x Dose	3.23	2	28	0.05	0.96

Note: This table presents main effects of subgroup and dose, and subgroup-by-dose interaction, with F statistics, p values, and effect size of Cohen d converted from partial eta squared.

b. Subgroup × Dose interaction contrasts: Pairwise comparisons

Group pairwise	Dose pairwise	Estimate	SE	95% CI	df	t	p	d
NTN_{A+} – NTN_{A-}	120 mg – placebo	-25.00	11.30	[-48.14, -1.86]	28	-2.21	0.04	-0.84
NTN_{A+} – NTN_{A-}	80 mg – placebo	-26.87	11.76	[-50.97, -2.78]	28	-2.28	0.03	-0.86
NTN_{A+} – NTN_{A-}	120 mg – 80 mg	1.87	10.73	[-20.1, 23.85]	28	0.17	0.86	0.07

Note: This table presents pairwise comparisons of dose effects between NTN_{A+} and NTN_{A-} subgroups, including estimated differences, SE, 95% CIs, t statistics, p values, and effect size of Cohen d.

c. Within-subgroup pairwise dose contrasts

NTN_{A+} dose contrast	Estimate	SE	95% CI	df	t	p	d
120 mg – placebo	8.75	7.99	[-7.62, 25.12]	28	1.10	0.28	0.42
80 mg – placebo	5.62	8.32	[-11.42, 22.66]	28	0.68	0.50	0.26
120 mg – 80 mg	3.12	7.59	[-12.43, 18.67]	28	0.41	0.68	0.15
**NTN_{A-} dose contrast**	**Estimate**	**SE**	**95% CI**	**df**	**t**	**p**	**d**
120 mg – placebo	33.75	7.99	[17.38, 50.12]	28	4.22	<0.001	1.60
80 mg – placebo	32.50	8.32	[15.46, 49.54]	28	3.91	0.001	1.48
120 mg – 80 mg	1.25	7.59	[-14.3, 16.8]	28	0.16	0.87	0.06

Note: This table presents within-subgroup pairwise dose contrasts separately for NTN_{A+} and NTN_{A-} subgroups, including estimated differences, SE, 95% CIs, t values, p values, and effect size of Cohen d.

d. Within-subgroup dose estimates

NTN_{A+} subgroup				NTN_{A-} subgroup			
**Dose**	**Estimate**	**SE**	**95% CI**	**Dose**	**Estimate**	**SE**	**95% CI**
Placebo	7.50	6.16	[-5.72, 20.72]	Placebo	-8.75	6.16	[-21.97, 4.47]
80 mg	13.13	5.63	[1.04, 25.21]	80 mg	23.75	5.63	[11.67, 35.83]
120 mg	16.25	5.13	[5.24, 27.26]	120 mg	25.00	5.13	[13.99, 36.01]

Note: This table presents estimated means for each dose condition within the NTN_{A+} and NTN_{A-} subgroups, including estimates, SE, and 95% CIs.

Abbreviations: df, Degree of freedom adjusted using the containment method; SE, Standard error; CI, confidence interval; d, Cohen d; NTN_{A+}, High negative affect circuit activity evoked by nonconscious threat; NTN_{A-}, Low negative affect circuit activity evoked by nonconscious threat.

© 2025 Zhang X et al. *JAMA Network Open*.

eTable 8. Summary of the Linear Mixed Model for VAS Feeling Secure

a. Model effects

Effect	F	Numerator df	Denominator df	p	d
Subgroup	0.24	1	14	0.63	0.26
Dose	7.17	2	28	0.00	1.43
Subgroup x Dose	2.81	2	28	0.08	0.90

Note: This table presents main effects of subgroup and dose, and subgroup-by-dose interaction, with F statistics, p values, and effect size of Cohen d converted from partial eta squared.

b. Subgroup × Dose interaction contrasts: Pairwise comparisons

Group pairwise	Dose pairwise	Estimate	SE	95% CI	df	t	p	d
NTN_{A+} – NTN_{A-}	120 mg – placebo	-20.00	9.23	[-38.90, -1.10]	28	-2.17	0.04	-0.82
NTN_{A+} – NTN_{A-}	80 mg – placebo	1.87	6.42	[-11.27, 15.02]	28	0.29	0.77	0.11
NTN_{A+} – NTN_{A-}	120 mg – 80 mg	-21.88	9.60	[-41.53, -2.22]	28	-2.28	0.03	-0.86

Note: This table presents pairwise comparisons of dose effects between NTN_{A+} and NTN_{A-} subgroups, including estimated differences, SE, 95% CIs, t statistics, p values, and effect size of Cohen d.

c. Within-subgroup pairwise dose contrasts

**NTN_{A+} dose contrast**	**Estimate**	**SE**	**95% CI**	**df**	**t**	**p**	**d**
120 mg – placebo	3.12	6.53	[-10.26, 16.5]	28	0.48	0.64	0.18
80 mg – placebo	11.25	4.54	[1.95, 20.55]	28	2.48	0.02	0.94
120 mg – 80 mg	-8.13	6.79	[-22.04, 5.78]	28	-1.20	0.24	-0.45
**NTN_{A-} dose contrast**	**Estimate**	**SE**	**95% CI**	**df**	**t**	**p**	**d**
120 mg – placebo	23.12	6.53	[9.74, 36.5]	28	3.54	0.001	1.34
80 mg – placebo	9.37	4.54	[0.07, 18.67]	28	2.07	0.05	0.78
120 mg – 80 mg	13.75	6.79	[-0.16, 27.66]	28	2.03	0.05	0.77

Note: This table presents within-subgroup pairwise dose contrasts separately for NTN_{A+} and NTN_{A-} subgroups, including estimated differences, SE, 95% CIs, t values, p values, and effect size of Cohen d.

d. Within-subgroup dose estimates

**NTN_{A+} subgroup**				**NTN_{A-} subgroup**			
**Dose**	**Estimate**	**SE**	**95% CI**	**Dose**	**Estimate**	**SE**	**95% CI**
Placebo	2.50	3.42	[-4.83, 9.83]	Placebo	-5.62	3.42	[-12.96, 1.71]
80 mg	13.75	3.89	[5.4, 22.1]	80 mg	3.75	3.89	[-4.6, 12.1]
120 mg	5.62	6.09	[-7.45, 18.7]	120 mg	17.50	6.09	[4.43, 30.57]

Note: This table presents estimated means for each dose condition within the NTN_{A+} and NTN_{A-} subgroups, including estimates, SE, and 95% CIs.

Abbreviations: df, Degree of freedom adjusted using the containment method; SE, Standard error; CI, confidence interval; d, Cohen d; NTN_{A+}, High negative affect circuit activity evoked by nonconscious threat; NTN_{A-}, Low negative affect circuit activity evoked by nonconscious threat.

© 2025 Zhang X et al. *JAMA Network Open*.

eTable 9. Summary of the Linear Mixed Model for Anxiety

a. Model effects

Effect	F	Numerator df	Denominator df	p	d
Subgroup	5.95	1	14	0.03	1.30
Dose	7.38	2	28	0.00	1.45
Subgroup x Dose	3.29	2	28	0.05	0.97

Note: This table presents main effects of subgroup and dose, and subgroup-by-dose interaction, with F statistics, p values, and effect size of Cohen d converted from partial eta squared.

b. Subgroup × Dose interaction contrasts: Pairwise comparisons

Group pairwise	Dose pairwise	Estimate	SE	95% CI	df	t	p	d
NTN_{A+} – NTN_{A-}	120 mg – placebo	8.44	3.44	[1.38, 15.49]	28	2.45	0.02	0.93
NTN_{A+} – NTN_{A-}	80 mg – placebo	2.44	3.18	[-4.07, 8.94]	28	0.77	0.45	0.29
NTN_{A+} – NTN_{A-}	120 mg – 80 mg	6.00	4.69	[-3.6, 15.6]	28	1.28	0.21	0.48

Note: This table presents pairwise comparisons of dose effects between NTN_{A+} and NTN_{A-} subgroups, including estimated differences, SE, 95% CIs, t statistics, p values, and effect size of Cohen d.

c. Within-subgroup pairwise dose contrasts

**NTN_{A+} dose contrast**	**Estimate**	**SE**	**95% CI**	**df**	**t**	**p**	**d**
120 mg – placebo	8.79	2.44	[3.79, 13.79]	28	3.61	0.001	1.36
80 mg – placebo	5.63	2.25	[1.02, 10.24]	28	2.51	0.02	0.95
120 mg – 80 mg	3.17	3.31	[-3.61, 9.95]	28	0.96	0.35	0.36
**NTN_{A-} dose contrast**	**Estimate**	**SE**	**95% CI**	**df**	**t**	**p**	**d**
120 mg – placebo	0.35	2.44	[-4.65, 5.35]	28	0.15	0.89	0.06
80 mg – placebo	3.19	2.25	[-1.42, 7.8]	28	1.42	0.17	0.54
120 mg – 80 mg	-2.83	3.31	[-9.61, 3.95]	28	-0.86	0.40	-0.33

Note: This table presents within-subgroup pairwise dose contrasts separately for NTN_{A+} and NTN_{A-} subgroups, including estimated differences, SE, 95% CIs, t values, p values, and effect size of Cohen d.

d. Within-subgroup dose estimates

**NTN_{A+} subgroup**				**NTN_{A-} subgroup**			
**Dose**	**Estimate**	**SE**	**95% CI**	**Dose**	**Estimate**	**SE**	**95% CI**
Placebo	2.40	0.98	[0.28, 4.51]	Placebo	0.92	0.98	[-1.19, 3.03]
80 mg	8.02	2.45	[2.76, 13.28]	80 mg	4.10	2.45	[-1.15, 9.36]
120 mg	11.19	2.63	[5.55, 16.82]	120 mg	1.27	2.63	[-4.36, 6.91]

Note: This table presents estimated means for each dose condition within the NTN_{A+} and NTN_{A-} subgroups, including estimates, SE, and 95% CIs.

Abbreviations: df, Degree of freedom adjusted using the containment method; SE, Standard error; CI, confidence interval; d, Cohen d; NTN_{A+}, High negative affect circuit activity evoked by nonconscious threat; NTN_{A-}, Low negative affect circuit activity evoked by nonconscious threat.

© 2025 Zhang X et al. *JAMA Network Open*.

eTable 10. Summary of the Linear Mixed Model for Impaired Control and Cognition

a. Model effects

Effect	F	Numerator df	Denominator df	p	d
Subgroup	9.14	1	14	0.01	1.68
Dose	8.69	2	28	0.00	1.58
Subgroup x Dose	3.82	2	28	0.03	1.04

Note: This table presents main effects of subgroup and dose, and subgroup-by-dose interaction, with F statistics, p values, and effect size of Cohen d converted from partial eta squared.

b. Subgroup × Dose interaction contrasts: Pairwise comparisons

Group pairwise	Dose pairwise	Estimate	SE	95% CI	df	t	p	d
NTN_{A+} – NTN_{A-}	120 mg – placebo	11.07	5.66	[-0.53, 22.67]	28	1.95	0.06	0.74
NTN_{A+} – NTN_{A-}	80 mg – placebo	9.66	4.94	[-0.46, 19.78]	28	1.95	0.06	0.74
NTN_{A+} – NTN_{A-}	120 mg – 80 mg	1.41	7.52	[-13.99, 16.81]	28	0.19	0.85	0.07

Note: This table presents pairwise comparisons of dose effects between NTN_{A+} and NTN_{A-} subgroups, including estimated differences, SE, 95% CIs, t statistics, p values, and effect size of Cohen d.

c. Within-subgroup pairwise dose contrasts

NTN_{A+} dose contrast	Estimate	SE	95% CI	df	t	p	d
120 mg – placebo	13.79	4.01	[5.58, 22]	28	3.44	0.002	1.30
80 mg – placebo	12.20	3.49	[5.05, 19.35]	28	3.49	0.002	1.32
120 mg – 80 mg	1.59	5.32	[-9.31, 12.49]	28	0.30	0.77	0.11
**NTN_{A-} dose contrast**	**Estimate**	**SE**	**95% CI**	**df**	**t**	**p**	**d**
120 mg – placebo	2.71	4.01	[-5.5, 10.92]	28	0.68	0.50	0.26
80 mg – placebo	2.54	3.49	[-4.61, 9.69]	28	0.73	0.47	0.28
120 mg – 80 mg	0.18	5.32	[-10.72, 11.08]	28	0.03	0.97	0.01

Note: This table presents within-subgroup pairwise dose contrasts separately for NTN_{A+} and NTN_{A-} subgroups, including estimated differences, SE, 95% CIs, t values, p values, and effect size of Cohen d.

d. Within-subgroup dose estimates

NTN_{A+} subgroup				NTN_{A-} subgroup			
**Dose**	**Estimate**	**SE**	**95% CI**	**Dose**	**Estimate**	**SE**	**95% CI**
Placebo	2.24	0.80	[0.51, 3.96]	Placebo	0.83	0.80	[-0.89, 2.56]
80 mg	14.44	3.58	[6.69, 22.18]	80 mg	3.37	3.58	[-4.38, 11.11]
120 mg	16.02	4.08	[7.2, 24.85]	120 mg	3.55	4.08	[-5.28, 12.37]

Note: This table presents estimated means for each dose condition within the NTN_{A+} and NTN_{A-} subgroups, including estimates, SE, and 95% CIs.

Abbreviations: df, Degree of freedom adjusted using the containment method; SE, Standard error; CI, confidence interval; d, Cohen d; NTN_{A+}, High negative affect circuit activity evoked by nonconscious threat; NTN_{A-}, Low negative affect circuit activity evoked by nonconscious threat.

© 2025 Zhang X et al. *JAMA Network Open*.

eTable 11. Summary of the Linear Mixed Model for Right Amygdala Activity With Multiple Imputation

a. Model effects

Effect	F	Numerator df	Denominator df	p	d
Subgroup	0.59	1	14	0.46	0.41
Dose	10.41	2	28	0.00	1.72
Subgroup x Dose	3.08	2	28	0.06	0.94

Note: This table presents main effects of subgroup and dose, and subgroup-by-dose interaction, with F statistics, p values, and effect size of Cohen d converted from partial eta squared.

b. Subgroup × Dose interaction contrasts: Pairwise comparisons

Group pairwise	Dose pairwise	Estimate	SE	95% CI	df	t	p	d
NTN_{A+} – NTN_{A-}	120 mg – placebo	-1.17	0.52	[-2.24, -0.1]	28	-2.26	0.03	-0.85
NTN_{A+} – NTN_{A-}	80 mg – placebo	-0.58	0.62	[-1.85, 0.69]	28	-0.94	0.36	-0.36
NTN_{A+} – NTN_{A-}	120 mg – 80 mg	-0.59	0.43	[-1.47, 0.29]	28	-1.38	0.18	-0.52

Note: This table presents pairwise comparisons of dose effects between NTN_{A+} and NTN_{A-} subgroups, including estimated differences, SE, 95% CIs, t statistics, p values, and effect size of Cohen d.

c. Within-subgroup pairwise dose contrasts

NTN_{A+} dose contrast	Estimate	SE	95% CI	df	t	p	d
120 mg – placebo	-1.11	0.37	[-1.87, -0.35]	28	-3.03	0.01	-1.15
80 mg – placebo	0.12	0.44	[-0.78, 1.02]	28	0.27	0.79	0.10
120 mg – 80 mg	-1.23	0.30	[-1.84, -0.62]	28	-4.06	0.00	-1.53
**NTN_{A-} dose contrast**	**Estimate**	**SE**	**95% CI**	**df**	**t**	**p**	**d**
120 mg – placebo	0.06	0.37	[-0.7, 0.82]	28	0.17	0.87	0.06
80 mg – placebo	0.70	0.44	[-0.2, 1.6]	28	1.60	0.12	0.60
120 mg – 80 mg	-0.64	0.30	[-1.25, -0.03]	28	-2.11	0.04	-0.80

Note: This table presents within-subgroup pairwise dose contrasts separately for NTN_{A+} and NTN_{A-} subgroups, including estimated differences, SE, 95% CIs, t values, p values, and effect size of Cohen d.

d. Within-subgroup dose estimates

NTN_{A+} subgroup				NTN_{A-} subgroup			
**Dose**	**Estimate**	**SE**	**95% CI**	**Dose**	**Estimate**	**SE**	**95% CI**
Placebo	0.31	0.34	[-0.42, 1.04]	Placebo	-0.11	0.34	[-0.84, 0.62]
80 mg	0.43	0.27	[-0.16, 1.01]	80 mg	0.59	0.27	[0.01, 1.17]
120 mg	-0.80	0.13	[-1.08, -0.52]	120 mg	-0.05	0.13	[-0.33, 0.23]

Note: This table presents estimated means for each dose condition within the NTN_{A+} and NTN_{A-} subgroups, including estimates, SE, and 95% CIs.

Abbreviations: df, Degree of freedom adjusted using the containment method; SE, Standard error; CI, confidence interval; d, Cohen d; NTN_{A+}, High negative affect circuit activity evoked by nonconscious threat; NTN_{A-}, Low negative affect circuit activity evoked by nonconscious threat.

© 2025 Zhang X et al. *JAMA Network Open*.

eTable 12. Summary of the Linear Mixed Model for sgACC Activity With Multiple Imputation

a. Model effects

Effect	F	Numerator df	Denominator df	p	d
Subgroup	0.23	1	14	0.64	0.26
Dose	3.52	2	28	0.04	1.00
Subgroup x Dose	1.19	2	28	0.32	0.58

Note: This table presents main effects of subgroup and dose, and subgroup-by-dose interaction, with F statistics, p values, and effect size of Cohen d converted from partial eta squared.

b. Subgroup × Dose interaction contrasts: Pairwise comparisons

Group pairwise	Dose pairwise	Estimate	SE	95% CI	df	t	p	d
NTN_{A+} – NTN_{A-}	120 mg – placebo	-0.80	0.73	[-2.3, 0.7]	28	-1.10	0.28	-0.42
NTN_{A+} – NTN_{A-}	80 mg – placebo	0.14	1.13	[-2.17, 2.45]	28	0.12	0.90	0.05
NTN_{A+} – NTN_{A-}	120 mg – 80 mg	-0.94	0.87	[-2.72, 0.84]	28	-1.08	0.29	-0.41

Note: This table presents pairwise comparisons of dose effects between NTN_{A+} and NTN_{A-} subgroups, including estimated differences, SE, 95% CIs, t statistics, p values, and effect size of Cohen d.

c. Within-subgroup pairwise dose contrasts

NTN_{A+} dose contrast	Estimate	SE	95% CI	df	t	p	d
120 mg – placebo	-0.83	0.51	[-1.87, 0.21]	28	-1.61	0.12	-0.61
80 mg – placebo	0.68	0.80	[-0.96, 2.32]	28	0.85	0.40	0.32
120 mg – 80 mg	-1.51	0.62	[-2.78, -0.24]	28	-2.45	0.02	-0.93
**NTN_{A-} dose contrast**	**Estimate**	**SE**	**95% CI**	**df**	**t**	**p**	**d**
120 mg – placebo	-0.03	0.51	[-1.07, 1.01]	28	-0.05	0.96	-0.02
80 mg – placebo	0.54	0.80	[-1.1, 2.18]	28	0.67	0.51	0.25
120 mg – 80 mg	-0.57	0.62	[-1.84, 0.7]	28	-0.92	0.37	-0.35

Note: This table presents within-subgroup pairwise dose contrasts separately for NTN_{A+} and NTN_{A-} subgroups, including estimated differences, SE, 95% CIs, t values, p values, and effect size of Cohen d.

d. Within-subgroup dose estimates

NTN_{A+} subgroup				NTN_{A-} subgroup			
**Dose**	**Estimate**	**SE**	**95% CI**	**Dose**	**Estimate**	**SE**	**95% CI**
Placebo	-0.06	0.53	[-1.2, 1.08]	Placebo	-0.08	0.53	[-1.22, 1.06]
80 mg	0.62	0.63	[-0.73, 1.97]	80 mg	0.46	0.63	[-0.89, 1.82]
120 mg	-0.89	0.14	[-1.18, -0.59]	120 mg	-0.10	0.14	[-0.4, 0.19]

Note: This table presents estimated means for each dose condition within the NTN_{A+} and NTN_{A-} subgroups, including estimates, SE, and 95% CIs.

Abbreviations: df, Degree of freedom adjusted using the containment method; SE, Standard error; CI, confidence interval; d, Cohen d; NTN_{A+}, High negative affect circuit activity evoked by nonconscious threat; NTN_{A-}, Low negative affect circuit activity evoked by nonconscious threat.

© 2025 Zhang X et al. *JAMA Network Open*.

eTable 13. Summary of the Linear Mixed Model for sgACC to Right Amygdala Connectivity With Multiple Imputation

a. Model effects

Effect	F	Numerator df	Denominator df	p	d
Subgroup	0.07	1	14	0.79	0.15
Dose	0.66	2	28	0.52	0.43
Subgroup x Dose	5.67	2	28	0.01	1.27

Note: This table presents main effects of subgroup and dose, and subgroup-by-dose interaction, with F statistics, p values, and effect size of Cohen d converted from partial eta squared.

b. Subgroup × Dose interaction contrasts: Pairwise comparisons

Group pairwise	Dose pairwise	Estimate	SE	95% CI	df	t	p	d
NTN_{A+} – NTN_{A-}	120 mg – placebo	0.54	0.20	[0.13, 0.95]	28	2.73	0.01	1.03
NTN_{A+} – NTN_{A-}	80 mg – placebo	-0.40	0.52	[-1.47, 0.67]	28	-0.78	0.44	-0.29
NTN_{A+} – NTN_{A-}	120 mg – 80 mg	0.94	0.48	[-0.04, 1.92]	28	1.97	0.06	0.74

Note: This table presents pairwise comparisons of dose effects between NTN_{A+} and NTN_{A-} subgroups, including estimated differences, SE, 95% CIs, t statistics, p values, and effect size of Cohen d.

c. Within-subgroup pairwise dose contrasts

**NTN_{A+} dose contrast**	Estimate	SE	95% CI	df	t	p	d
120 mg – placebo	0.25	0.14	[-0.04, 0.54]	28	1.78	0.09	0.67
80 mg – placebo	-0.49	0.37	[-1.25, 0.27]	28	-1.35	0.19	-0.51
120 mg – 80 mg	0.74	0.34	[0.04, 1.44]	28	2.19	0.04	0.83
**NTN_{A-} dose contrast**	**Estimate**	**SE**	**95% CI**	**df**	**t**	**p**	**d**
120 mg – placebo	-0.29	0.14	[-0.58, 0]	28	-2.08	0.05	-0.79
80 mg – placebo	-0.09	0.37	[-0.85, 0.67]	28	-0.25	0.81	-0.09
120 mg – 80 mg	-0.20	0.34	[-0.9, 0.5]	28	-0.59	0.56	-0.22

Note: This table presents within-subgroup pairwise dose contrasts separately for NTN_{A+} and NTN_{A-} subgroups, including estimated differences, SE, 95% CIs, t values, p values, and effect size of Cohen d.

d. Within-subgroup dose estimates

NTN_{A+} subgroup				NTN_{A-} subgroup			
**Dose**	**Estimate**	**SE**	**95% CI**	**Dose**	**Estimate**	**SE**	**95% CI**
Placebo	0.40	0.21	[-0.05, 0.85]	Placebo	0.37	0.21	[-0.08, 0.81]
80 mg	-0.10	0.37	[-0.9, 0.7]	80 mg	0.28	0.37	[-0.52, 1.08]
120 mg	0.65	0.16	[0.31, 0.98]	120 mg	0.07	0.16	[-0.26, 0.41]

Note: This table presents estimated means for each dose condition within the NTN_{A+} and NTN_{A-} subgroups, including estimates, SE, and 95% CIs.

Abbreviations: df, Degree of freedom adjusted using the containment method; SE, Standard error; CI, confidence interval; d, Cohen d; NTN_{A+}, High negative affect circuit activity evoked by nonconscious threat; NTN_{A-}, Low negative affect circuit activity evoked by nonconscious threat.

© 2025 Zhang X et al. *JAMA Network Open*.

eTable 14. Summary of Participants' Neural, Behavioral, and Affective Changes and Quotations After Administration of 120 mg of MDMA vs Placebo

Participants	Group	Δ Amygdala R activity	Δ sgACC activity	Δ sgACC-Amygdala R connectivity	Δ Likability of threat faces	Δ Wanting to be with other people	Δ Feeling secure	Δ Anxiety	Δ Impaired control and cognition	Quotes
P001	NTN$_{A-}$	1.05	0.65	-0.67	5	15	0	2.83	3.71	*"This is what molly feels like, this is a positive experience."*
P002	NTN$_{A-}$	-1.73	-0.68	-0.20	0	30	40	1.83	9.86	*"I want to hug all my friends and snuggle up with someone."*
P003	NTN$_{A-}$	-0.92	0.40	0.71	0	40	10	0.17	-0.14	*"I am feeling a little overwhelmed…it is hard to focus [and] that is making me feel a little insecure…I like to have control over my body."*
P005	NTN$_{A+}$	-1.97	-0.34	0.27	30	20	25	4.17	2.00	*"[MDMA] shows you where you want to get to, this is the destination you want. It is kind of a shortcut, it is kind of helping you find your path in the woods… if you're lost, there is the destination."*

© 2025 Zhang X et al. *JAMA Network Open*.

eTable 14. Summary of participants' neural, behavioral, and affective changes, and quotes after administration of 120 mg MDMA versus placebo (continued)

P006	NTN_{A+}	-0.04	1.60	-0.52	20	10	0	0.00	8.00	*"I feel slightly less emotionally secure than I did earlier this morning... I had this feeling of incredible calm from the weekend and now I don't quite feel that anymore."*
P007	NTN_{A+}	-0.84	-1.68	-0.02	10	20	0	4.33	4.29	*"This is difficult, putting sentences together."*
P008	NTN_{A-}	1.00	-2.54	-0.66	0	100	50	-2.17	-2.43	*"I needed to try and be as normal as possible but […] why am I judging myself for like trying to act like the drug isn't having an effect on me."*
P009	NTN_{A+}	-0.70	-1.08	0.35	0	10	-10	2.67	1.29	*"I felt like all of the emotions were fair game, but uh, it would be accepting of all of them if it came up […] and so I'm grateful for that you know when, if I think of something as negative, I think of it as something to learn from, and I don't think of it as something to avoid per se […] it was like a willingness to accept and confront things."*

© 2025 Zhang X et al. *JAMA Network Open*.

eTable 14. Summary of participants' neural, behavioral, and affective changes, and quotes after administration of 120 mg MDMA versus placebo (continued)

Participants	Group	Δ Amygdala R activity	Δ sgACC activity	Δ sgACC-Amygdala R connectivity	Δ Likability of threat faces	Δ Wanting to be with other people	Δ Feeling secure	Δ Anxiety	Δ Impaired control and cognition	Quotes
P010	NTN_{A-}	-1.29	0.23	-1.02	0	30	15	2.33	2.43	*"I just feel like super present and uhm, yeah there's no confusion about anything. I just feel like really sort of perfectly aware."*
P011	NTN_{A+}	-2.80	-3.84	0.06	30	30	10	14.83	28.29	*"It seemed like at the time like everything had like, this extraordinary meaning."*
P012	NTN_{A-}	1.48	0.96	-0.19	-20	0	40	-0.50	-0.43	*"I am having a wonderful time… I'm just having a really good time; I just don't want it to end."*
P013	NTN_{A-}	0.65	0.84	-0.09	10	20	-10	-1.33	6.86	*"It feels like […] everything is lighter, like when you're walking your feet kind of float up."*

© 2025 Zhang X et al. *JAMA Network Open*.

eTable 14. Summary of participants' neural, behavioral, and affective changes, and quotes after administration of 120 mg MDMA versus placebo (continued)

Participants	Group	Δ Amygdala R activity	Δ sgACC activity	Δ sgACC-Amygdala R connectivity	Δ Likability of threat faces	Δ Wanting to be with other people	Δ Feeling secure	Δ Anxiety	Δ Impaired control and cognition	Quotes
P014	NTN_{A+}	-0.83	-1.23	0.68	0	0	-20	24.83	25.00	*"There were some valleys, but […] I feel like if you're in the right mental health, you're able to overcome… pass through those valleys and darkness and come up to that hill and just see how beautiful things are."*
P015	NTN_{A+}	-0.11	2.99	-0.63	0	30	0	0.00	0.86	*"I'm saying silly happy because it's just like a little lighter than I was before. I feel the mood shifting, like I'm smiling in a goofy way."*
P016	NTN_{A-}	0.30	-2.29	-0.77	-20	35	40	-0.33	1.86	*"I feel like in the other time I was like more in my head and having really like more positive loving thoughts, which I'm having now too, but I'm really loving how my body feels and really enjoying."*

© 2025 Zhang X et al. *JAMA Network Open*.

eTable 14. Summary of participants' neural, behavioral, and affective changes, and quotes after administration of 120-mg MDMA versus placebo (continued)

P017	NTN_{A+}	-0.50	-1.52	-0.43	0	-50	20	19.50	40.57	*"I feel amazing. The pleasant physical sensations are overwhelming in a good way. I'm in an amazing mood and have no worries."*

Note: Each measure is quantified as the change (Δ) induced by 120 mg of MDMA relative to placebo condition from multiple imputations. Quotes are selected from the 120-mg MDMA condition.

Abbreviations: MDMA, 3,4-methylenedioxymethamphetamine; NTN_{A+}, High negative affect circuit activity evoked by nonconscious threat; NTN_{A-}, Low negative affect circuit activity evoked by nonconscious threat; R, Right; sgACC, Subgenual anterior cingulate cortex.

© 2025 Zhang X et al. *JAMA Network Open*.

eTable 15. Accuracy of Dose Identification Across Different Drug Conditions

**Group**	**Placebo**	**80 mg**	**120 mg**
	**No. [%]**	**No. [%]**	**No. [%]**
All participants	14[88]	9[64]	11[79]
NTN_{A+}	6[75]	5[71]	6[100]
NTN_{A-}	8[100]	4[57]	5[63]
Study clinicians	15[94]	11[73]	13[87]
Research coordinators	13[87]	12[75]	13[87]

Abbreviations: NTN_{A+}, High negative affect circuit activity evoked by nonconscious threat; NTN_{A-}, Low negative affect circuit activity evoked by nonconscious threat.

© 2025 Zhang X et al. *JAMA Network Open*.

eFigure 1. Study Design and Baseline Stratification for the Randomized Controlled Mechanistic MDMA Trial

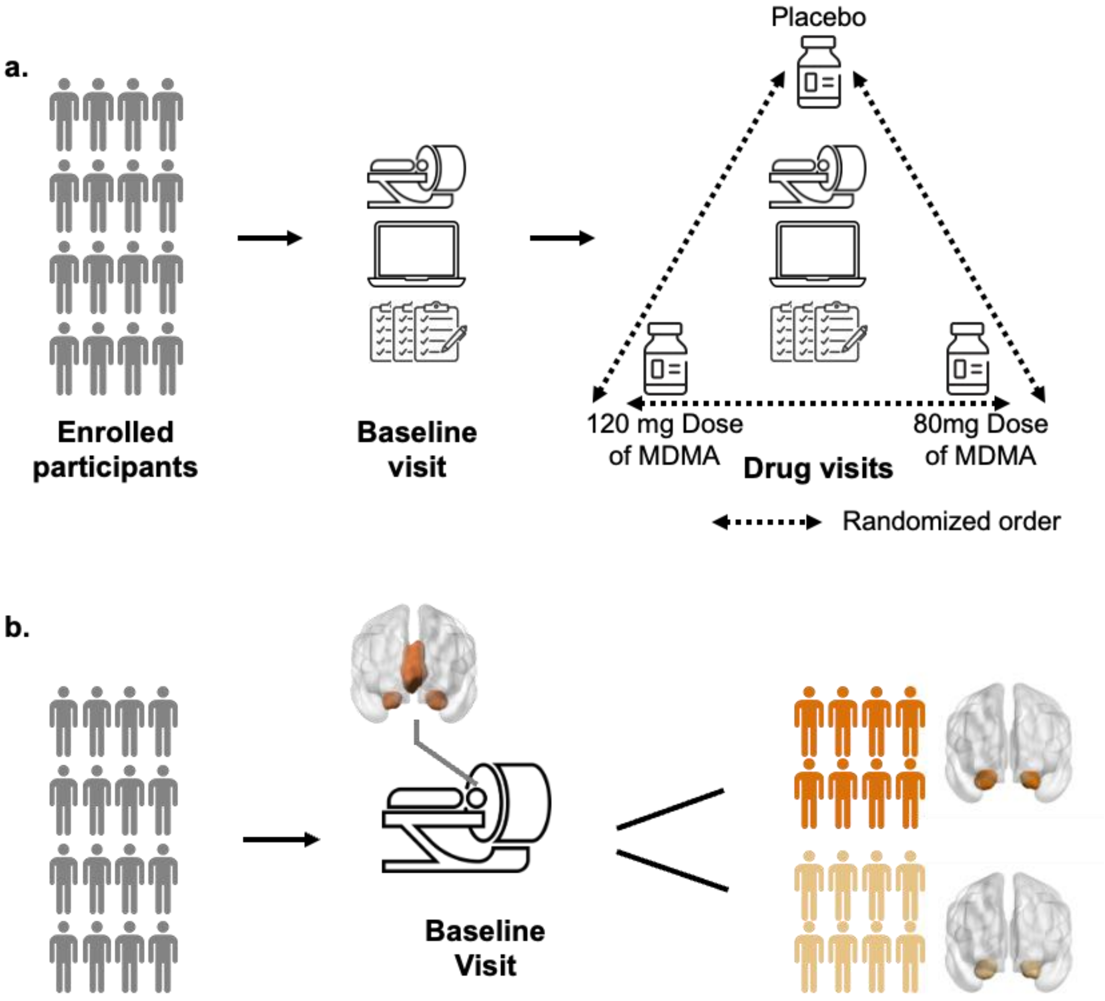

a. In a double-blinded, within-participants, placebo-and-baseline-controlled randomized design, 17 adult nonclinical participants were recruited, of which 16 participants completed all study visits. Each participant first completed a baseline visit, during which functional Magnetic Resonance Imaging (fMRI) scans, behavioral tests, and self-reports were acquired. Starting two days after the baseline visit, each participant received each condition (placebo, 80-mg MDMA, and 120-mg MDMA) in a randomized order, with 10-14 days between each visit to avoid drug carry-over effects. Under the acute effects of the drug in each condition, participants completed post-administration fMRI scans, behavioral tests, and self-reports. This study design enables participant comparisons to evaluate the effects of MDMA on neural, behavioral, and affective measures by ensuring each participant experiences all conditions in a randomized sequence. **b.** Each participant was assessed by the negative affect circuit during a facial expressions of emotion (FEET) fMRI task, in which participants nonconsciously viewed facial expressions of emotion. Activity of the amygdala in response to threat faces relative to neutral faces was quantified and was expressed in standard deviation units relative to a separate healthy reference dataset (z-scores). A median split was used to stratify all participants into high (NTN_{A+}; N = 8) and low (NTN_{A-}; N = 8) negative affect circuit activity subgroups.
Abbreviations: MDMA, 3,4-methylenedioxymethamphetamine; NTN_{A+}, High negative affect circuit activity evoked by nonconscious threat; NTN_{A-}, Low negative affect circuit activity evoked by nonconscious threat.

© 2025 Zhang X et al. *JAMA Network Open*.

eFigure 2. Baseline Clinical Symptoms for High (NTN_{A+}) and Low (NTN_{A-}) Negative Affect Circuit Activity Subgroups Evoked by Nonconscious Threat

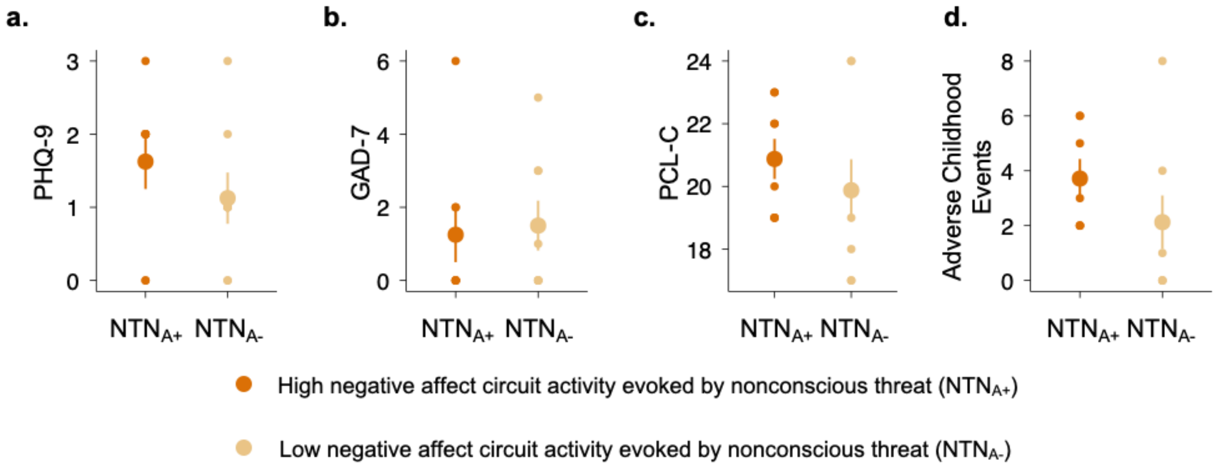

Baseline clinical symptoms of PHQ-9 (**a.**), GAD-7 (**b.**), PCL-C **(c.**), and Adverse Childhood Events measured by the Early Life Stress Questionnaire (**d.**) for NTN_{A+} and NTN_{A-} subgroups. In all plots, the big solid dots indicate the mean; small dots, individual data points; and error bars, SEM.

Abbreviations: GAD-7, 7-Item Generalized Anxiety Disorder; NTN_{A+}, High negative affect circuit activity evoked by nonconscious threat; NTN_{A-}, Low negative affect circuit activity evoked by nonconscious threat; PCL-C, Post-Traumatic Stress Disorder Checklist-Civilian Version; PHQ-9, 9-item Patient Health Questionnaire.

© 2025 Zhang X et al. *JAMA Network Open*.

eFigure 3. Acute Neural, Behavioral, and Affective Response to Placebo and MDMA at 80 mg and 120 mg for High (NTN_{A+}) and Low ($NTN_{A–}$) Negative Affect Circuit Activity Subgroups Evoked by Nonconscious Threat

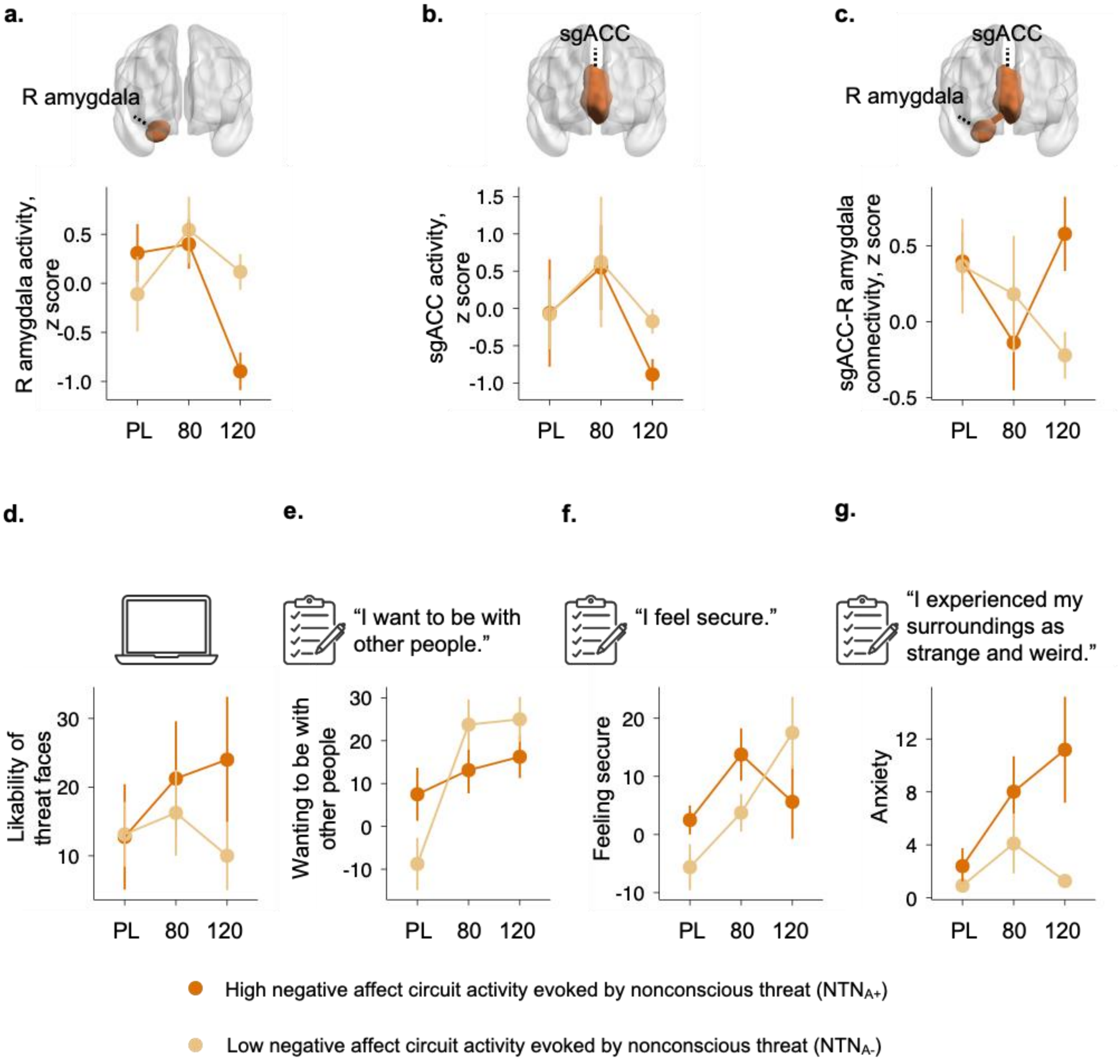

Right (R) amygdala activity (**a.**), subgenual anterior cingulate cortex (sgACC) activity (**b.**), functional connectivity between sgACC and right amygdala (**c.**), likability of threat faces (**d.**), wanting to be with other people (**e.**), feeling secure (**f.**), and anxiety (**g.**). In all plots, solid dots indicate the mean; error bars, SEM.

Abbreviation: MDMA, 3,4-methylenedioxymethamphetamine; NTN_{A+}, High negative affect circuit activity evoked by nonconscious threat; NTN_{A-}, Low negative affect circuit activity evoked by nonconscious threat; PL, Placebo; 80, 80-mg MDMA; 120, 120-mg MDMA.

© 2025 Zhang X et al. *JAMA Network Open*.

eReferences.

1. Bedi G, Phan KL, Angstadt M, de Wit H. Effects of MDMA on sociability and neural response to social threat and social reward. *Psychopharmacology (Berl)*. Nov 2009;207(1):73-83. doi:10.1007/s00213-009-1635-z
2. Korgaonkar MS, Grieve SM, Etkin A, Koslow SH, Williams LM. Using standardized fMRI protocols to identify patterns of prefrontal circuit dysregulation that are common and specific to cognitive and emotional tasks in major depressive disorder: first wave results from the iSPOT-D study. *Neuropsychopharmacol*. Apr 2013;38(5):863-71. doi:10.1038/npp.2012.252
3. Williams LM, Liddell BJ, Rathjen J, et al. Mapping the time course of nonconscious and conscious perception of fear: an integration of central and peripheral measures. *Hum Brain Mapp*. Feb 2004;21(2):64-74. doi:10.1002/hbm.10154
4. Goldstein-Piekarski AN, Ball TM, Samara Z, et al. Mapping Neural Circuit Biotypes to Symptoms and Behavioral Dimensions of Depression and Anxiety. *Biol Psychiatry*. Mar 15 2022;91(6):561-571. doi:10.1016/j.biopsych.2021.06.024
5. Goldstein-Piekarski AN, Wielgosz J, Xiao L, et al. Early changes in neural circuit function engaged by negative emotion and modified by behavioural intervention are associated with depression and problem-solving outcomes: A report from the ENGAGE randomized controlled trial. *Ebiomedicine*. May 2021;67:103387. doi:10.1016/j.ebiom.2021.103387
6. Yarkoni T, Poldrack RA, Nichols TE, Van Essen DC, Wager TD. Large-scale automated synthesis of human functional neuroimaging data. *Nat Methods*. Jun 26 2011;8(8):665-70. doi:10.1038/nmeth.1635
7. Tzourio-Mazoyer N, Landeau B, Papathanassiou D, et al. Automated anatomical labeling of activations in SPM using a macroscopic anatomical parcellation of the MNI MRI single-subject brain. *Neuroimage*. Jan 2002;15(1):273-89. doi:10.1006/nimg.2001.0978
8. Tozzi L, Zhang X, Pines A, et al. Personalized brain circuit scores identify clinically distinct biotypes in depression and anxiety. *Nature Medicine*. 2024/07/01 2024;30(7):2076-2087. doi:10.1038/s41591-024-03057-9
9. Williams LM, Mathersul D, Palmer DM, Gur RC, Gur RE, Gordon E. Explicit identification and implicit recognition of facial emotions: I. Age effects in males and females across 10 decades. *Journal of Clinical and Experimental Neuropsychology*. 2009/04/01 2009;31(3):257-277. doi:10.1080/13803390802255635
10. Dittrich A, Lamparter D, Maurer M. *5D-ABZ: Fragebogen zur Erfassung Aussergewöhnlicher Bewusstseinszustände. Eine kurze Einführung [5D-ASC: Questionnaire for the assessment of altered states of consciousness. A short introduction]*. PSIN PLUS; 2006.
11. Dittrich A, Lamparter D, Maurer M. *5D-ASC: Questionnaire for the assessment of altered states of consciousness. A Short Introduction*. 3rd edn ed. PSIN PLUS; 2010.
12. Bourgeois A, LeUnes A, Meyers M. Full-Scale and Short-Form of the Profile of Mood States: A Factor Analytic Comparison. Article. *Journal of Sport Behavior*. 2010;33(4):355-376.
13. Dolder PC, Muller F, Schmid Y, Borgwardt SJ, Liechti ME. Direct comparison of the acute subjective, emotional, autonomic, and endocrine effects of MDMA, methylphenidate, and modafinil in healthy subjects. *Psychopharmacology (Berl)*. Feb 2018;235(2):467-479. doi:10.1007/s00213-017-4650-5
14. Studerus E, Gamma A, Vollenweider FX. Psychometric evaluation of the altered states of consciousness rating scale (OAV). *PLoS One*. Aug 31 2010;5(8):e12412. doi:10.1371/journal.pone.0012412

© 2025 Zhang X et al. *JAMA Network Open*.

15. van Buuren S, Groothuis-Oudshoorn K. mice: Multivariate Imputation by Chained Equations in R. *Journal of Statistical Software*. 12/12 2011;45(3):1 - 67. doi:10.18637/jss.v045.i03
16. Williams LM, Korgaonkar MS, Song YC, et al. Amygdala Reactivity to Emotional Faces in the Prediction of General and Medication-Specific Responses to Antidepressant Treatment in the Randomized iSPOT-D Trial. *Neuropsychopharmacol*. Sep 2015;40(10):2398-408. doi:10.1038/npp.2015.89
17. Gur RC, Sara R, Hagendoorn M, et al. A method for obtaining 3-dimensional facial expressions and its standardization for use in neurocognitive studies. *Journal of Neuroscience Methods*. 2002/04/15/ 2002;115(2):137-143. doi:https://doi.org/10.1016/S0165-0270(02)00006-7
18. Mathersul D, Palmer DM, Gur RC, et al. Explicit identification and implicit recognition of facial emotions: II. Core domains and relationships with general cognition. *J Clin Exp Neuropsychol*. Apr 2009;31(3):278-91. doi:10.1080/13803390802043619

© 2025 Zhang X et al. *JAMA Network Open*.
