# Supplementary material for: Negative Affect Circuit Subtypes and Neural, Behavioral, and Affective Responses to MDMA: A Randomized Clinical Trial
Source: JAMA Netw Open. 2025 Apr 30;8(4):e257803. doi: 10.1001/jamanetworkopen.2025.7803 (PMC12044494; doi:10.1001/jamanetworkopen.2025.7803)
Supplement: Supplement 3. — Data Sharing Statement [file jamanetwopen-e257803-s003.pdf]

Data Sharing Statement

Zhang. Negative Affect Circuit Subtypes and Neural, Behavioral, and Affective Responses to MDMA. *JAMA Netw Open*. Published April 30, 2025. doi:10.1001/jamanetworkopen.2025.7803

Data
Additional Information: Stanford Regulating Circuits of the Brain Study - MDMA (RBRAIN-MDMA) https://clinicaltrials.gov/study/NCT04060108 NCT04060108
Data available: Yes
Data types: Deidentified participant data
How to access data: The study dataset is available upon request from Stanford BRAINnet at www.stanfordpmhw.com/datasets. The BRAINnet repository meets the requirements for being public but also aligns with the procedures of other official public and scientific repositories such as HCP, ABCD, and NDA. This choice aligns with the FAIRness guidelines and respects the original funding requirements, allowing for appropriate source contributions and citations. Our approach is specifically designed for scientific use, which includes limiting access to for-profit entities to comply with the original funding stipulations and participant consent. Therefore, total open access is not feasible. We intend to provide public access that is consistent with the consent agreements and the original funding intentions, similar to the data shared through NIH repositories. On Stanford BRAINnet, we established a data access request form that screens users, similar to other public repositories.
When available: With publication

Supporting Documents
Document types: Statistical/analytic code, Other (please specify)
Additional Information: Trial protocol - Supplement 1
How to access documents: The full analysis codes are available at https://github.com/WilliamsPanLab/MDMA-Amygdala-subtype. The trial protocol is attached as Supplement 1.
When available: With publication

Additional Information
Who can access the data: Researchers whose proposed use of the data has been approved
Types of analyses: For a specified approved purpose
Mechanisms of data availability: After approval of a proposal
